# Mitigation of atrial fibrillation‐related complications with antithrombotic and cytoreductive therapy in patients with Myeloproliferative Neoplasms: Implications from the GSG‐MPN bioregistry

**DOI:** 10.1002/hem3.70090

**Published:** 2025-03-17

**Authors:** Hyunyee Rosa Cho, Susanne Isfort, Kim Kricheldorf, Frank Stegelmann, Martine Klausmann, Florian H. Heidel, Martin Griesshammer, Holger Schulz, Andreas Hochhaus, Joachim Göthert, Rudolf Schlag, Wiebke Hollburg, Lino Teichmann, Katja Sockel, Stefan Wilop, Deniz Gezer, Martin Kirschner, Konstanze Döhner, Tim H. Brümmendorf, Steffen Koschmieder, for the German Study Group for Myeloproliferative Neoplasms (GSG‐MPN)

**Affiliations:** ^1^ Department of Hematology, Oncology, Hemostaseology, and Stem Cell Transplantation, Faculty of Medicine RWTH Aachen University, and Center for Integrated Oncology Aachen Bonn Cologne Düsseldorf (CIO ABCD) Aachen Germany; ^2^ Hematology, Hemostasis, Oncology and Stem Cell Transplantation, Hannover Medical School (MHH) Hannover Germany; ^3^ Department of Internal Medicine III University Hospital Ulm Ulm Germany; ^4^ Gemeinschaftspraxis, Aschaffenburg Aschaffenburg Germany; ^5^ Internal Medicine C University Medicine Greifswald Greifswald Germany; ^6^ University Clinic for Hematology, Oncology, Haemostaseology and Palliative Care, Johannes Wesling Medical Center Minden University of Bochum Bochum Germany; ^7^ Practice for Clinical Hematology and Oncology Frechen Germany; ^8^ Department of Hematology and Oncology Jena University Hospital Jena Germany; ^9^ Department of Hematology, West German Cancer Center (WTZ) University Hospital Essen Essen Germany; ^10^ Hämatologisch‐Onkologische Schwerpunktpraxis Würzburg Würzburg Germany; ^11^ HOPA‐Hämatologisch‐Onkologische Praxis Altona Hamburg Germany; ^12^ Department of Medicine III University Hospital Bonn, and Center for Integrated Oncology Aachen Bonn Cologne Düsseldorf (CIO ABCD) Bonn Germany; ^13^ Medical Clinic and Policlinic I University Hospital Dresden, TU Dresden Dresden Germany; ^14^ MVZ West GmbH Würselen, Hämatologie‐Onkologie Würselen Germany

Vascular complications such as thromboembolic events (TEs) and severe bleeding events (BEs) are the major causes of morbidity and mortality in patients (pts) with Myeloproliferative Neoplasms (MPNs).^1^ TEs are promoted by the hypercoagulable state in MPN, caused by elevated blood counts, activation of platelets, leukocytes, and endothelial cells, the presence of the JAK2V617F mutation, and increased circulating procoagulant microparticles and the occurrence of acquired activated protein C resistance. Disease‐associated bleeding may occur via acquired von Willebrand syndrome, platelet dysfunction, antiplatelet agents (APAs), and thrombocytopenia.^2^ In addition, hemostasis can be disturbed by anticoagulation therapy, acquired hemophilia,^3^ or complications such as liver dysfunction or disseminated intravascular coagulopathies due to infection.^2^

Atrial fibrillation (AF) is the most common sustained arrhythmia, with increasing prevalence with age.^4^, ^5^ The incidence of AF has rarely been studied in MPN, but was suggested to be higher compared to the general population.^6^ The same study found that MPN pts with AF had a higher frequency of cardiovascular risk factors (CVRFs) and thrombotic complications and a shorter thrombosis‐free survival than MPN pts without AF.^6^ However, in a separate study comparing 63 pts with polycythemia vera (PV) and AF to 124 control pts with AF only, no increased incidence of thrombosis was found.^7^ Both studies found no increase in major BE. However, none of the two studies reported the use and effects of cytoreductive therapy (CRT) in their cohorts, a critical means to decrease the risk of both initial and recurrent thrombosis in high‐risk MPN pts.

Therefore, we conducted this retrospective analysis of 2,780 MPN pts enrolled in the GSG‐MPN bioregistry, in order to identify the characteristics of AF in MPN pts and to assess the benefit–risk profile of antithrombotic therapies (ATTs) alone or in combination with MPN‐specific CRT. The German Study Group MPN bioregistry (GSG‐MPN bioregistry) is an ambispective observational study of MPN pts, with over 70 centers participating. Recruitment started in August 2012, with a data cut‐off date of January 2020.

As in the general population,^6^ the median age of AF pts in our MPN cohort was higher than those without AF (Supporting Information S1: Table S3). The prevalence of AF in pts older than 80 years of age in our MPN cohort was higher than that in the general population (18.2% [Supporting Information S1: Figure S1] vs. 8.8% in the FRAMINGHAM study^8^).

Since the incidence and complication rate of MPN pts increase with age and since CRT is recommended in an age‐adapted manner, we performed a case–control analysis following a 1:1 pattern, with age as a matching factor (Table 1, *n* = 134 with AF vs. *n* = 134 without AF). Here, unlike in non‐age‐matched analyses (Supporting Information S1: Table S3), which showed higher incidence of TEs and BEs in AF pts, no significant difference in the prevalence of TEs and BEs was observed after age‐matching. Also, the higher percentage of arterial TE in AF pts was no longer observed after age‐matching, suggesting that all pts (MPN pts as well as non‐MPN pts) develop a preponderance of arterial over venous TE with increasing age, as has been described before.^1^, ^9^, ^10^

**Table 1:** Comparisons of pts with versus without atrial fibrillation with age‐matched data.

			With AF^a^ (*n* = 134)			Without AF^a^ (*n* = 134)				
Variables			*N*	% column	Median (IQR)	*N*	% column	Median (IQR)	P	
Age (*n* = 268)	<65		18	13.4	76.0 (70–81)	18	13.4	75.9 (70–81)	0.987 >0.999	b
	65–74		36	26.9		37	27.6			
	>74		80	59.7		79	59.0			
Sex (*n* = 268)	Men		74	55.2		59	44.0		0.087	b
	Women		60	44.8		75	55.6			
MPN subtype^c^ (*n* = 265)	PV		42	31.3		42	32.1		0.136	b
	ET		41	30.6		44	33.6			
	PMF		27	20.1		33	25.2			
	PostPV‐MF		6	4.5		2	1.5			
	PostET‐MF		2	1.5		4	3.1			
	MPN‐ U		11	8.2		6	4.6			
	Others		5	3.7		0	0.0			
Leukocyte count (*n* = 265)	<3.5/nL		2	1.5	9.4 (8–14)	4	3.1	9.67 (7–13)	0.373 0.547	b
	3.5–10/nL		74	55.2		66	50.4			
	>10/nL		58	43.3		61	46.6			
Hematocrit (*n* = 261)	≤45/nL		80	60.6	42.5 (36–48)	88	68.2	42.8 (34–47)	0.434 0.245	b
	>5/nL		52	39.4		41	31.8			
Platelet count (*n* = 264)	<150/nL		11	8.2	451.0 (296–697)	16	12.3	501.5 (331–677)	0.791 0.261	b
	150–450/nL		55	41.0		42	32.3			
	>450/nL		68	50.7		72	55.4			
LDH (*n* = 235)	≤250 U/L		43	35.0	311.0 (232–407)	29	25.9	328.9 (245–455)	0.198 0.157	d b
	>250 U/L		80	65.0		83	74.1			
Myocardial infarction (*n* = 242)	Yes		10	7.9		11	9.6		0.655	b
	No^e^		117	92.1		104	90.4			
Congestive heart failure (*n* = 244)	Yes		28	21.9		9	7.8		0.002	b ^,^ *
	No^e^		100	78.1		107	92.2			
Diabetes mellitus (*n* = 268)	Yes		28	20.9		12	9.0		0.009	b ^,^ d
	No^e^		106	79.1		122	91.0			
Hypertension (*n* = 238)	Yes		90	72.6		69	60.5		0.054	b ^,^ #
	No^d^		34	27.4		45	39.5			
Splenomegaly (*n* = 237)	Yes		63	52.5		69	59.0		0.360	b
	No^d^		57	47.5		48	41.0			
CHA2DS2‐VASc (*n* = 268)	*M* ≤ 1, *F* ≤ 2		17	12.7	3.0 (2–4)	32	23.9	3.0 (2–4)	0.025 0.026	* b ^,^ *
	*M* > 1, *F* > 2		117	87.3		102	76.1			
HAS‐BLED (*n* = 268)	≤2		74	55.2	2.0 (2–3)	105	78.4	2.0 (1–2)	<0.001 <0.001	* b ^,^ *
	>2		60	44.8		29	21.6			
TE at or after diagnosis (*n* = 268)	Yes		21	15.7		17	12.7		0.600	b
	No^d^		113	84.3		117	87.3			
TE regardless of time (*n* = 251)	Yes		63	48.5		70	57.9		0.164	b
	No		67	51.5		51	42.1			
Types of TE (*n* = 118)	Arterial		46	68.7		34	66.7		0.960	b
	Venous		13	19.4		10	19.6			
	Both		8	11.9		7	13.7			
Bleeding at or after diagnosis (*n* = 268)	Yes		11	8.2		6	4.5		0.316	b
	No		123	91.8		128	95.5			
Bleeding event regardless of time (*n* = 250)	Yes		13	10.2		8	6.5		0.364	b
	No^d^		114	89.8		115	93.5			
Antithrombotic therapy (*n* = 268)	Antiplatelet therapy	Yes	51	38.1		78	58.2		0.001	b ^,^ *
		No	83	61.9		56	41.8			
	VKA	Yes	47	35.1		10	7.5		<0.001	b ^,^ *
		No	87	64.9		124	92.5			
	DOAK	Yes	50	37.3		12	9.0		<0.001	b ^,^ *
		No	84	62.7		122	91.0			
	Heparin	Yes	6	4.5		3	2.2		0.500	b
		No	128	95.5		131	97.8			
	Any type of antithrombotic^e^	Yes	115	85.8		92	68.7		0.001	b ^,^ *
		No	19	14.2		42	31.3			
MPN therapy (*n* = 268)	Anagrelide	Yes	23	17.2		21	15.7		0.869	b
		No	111	82.8		113	84.3			
	HU	Yes	86	64.2		74	55.2		0.171	b
		No	48	35.8		60	44.8			
	Ruxolitinib	Yes	35	26.1		49	36.6		0.087	b ^,^ *
		No	99	73.9		85	63.4			
	Interferon	Yes	13	9.7		12	9.0		>0.999	b
		No	121	90.3		122	91.0			
	Imide	Yes	3	2.2		4	3.0		>0.999	b
		No	131	97.8		130	97.0			
	Any type of MPN Therapy^e^	Yes	102	76.1		105	78.4		0.771	b
		No	32	23.9		29	21.6			
Driver mutations	JAK2 (*n* = 249)	Yes	94	74.6		103	83.7		0.087	b ^,^ #
		No	32	25.4		20	16.3			
	CalR (*n* = 98)	Yes	9	18.8		10	20.0		>0.999	b
		No	39	81.3		40	80.0			
	MPL (*n* = 93)	Yes	6	12.2		5	11.4		>0.999	b
		No	43	87.8		39	88.6			

Abbreviations: AF, atrial fibrillation; LDH, lactate dehydrogenase; TE, thromboembolic events.

^a^ Patients with a history of successful cardioversion before the date of MPN diagnosis were not considered to have AF.

^b^ Fisher's Exact test for categorized variables.

^c^ Polycythemia vera (PV), essential thrombocythemia (ET), primary myelofibrosis (PMF), MPN unclassifiable (MPN‐U), postPV‐myelofibrosis (postPV‐MF), post‐ET myelofibrosis (postET‐MF).

^d^ No or not documented.

^e^ Any type of therapy; these pts could have been treated with several therapies, either simultaneously or separately.

* *P* < 0.05.

# Borderline significance *P* < 0.09.

While cardiovascular comorbidities are reported to be risk factors for the development of AF,^8^, ^11^ in our age‐matched cohort, it was revealed that AF pts had more cardiac comorbidities than non‐AF pts. In addition, use of any type of ATT, including vitamin K antagonists (VKAs) and direct oral anticoagulant (DOAC), was more prominent in AF pts. Conversely, a higher percentage of non‐AF pts were treated with APA.

When analyzing the entire cohort of MPN pts without age‐matching, our Kaplan–Meier survival analyses showed significantly inferior OS in pts with coexisting AF (Supplemental Figure 4A). 5‐year OS was 92% in pts without AF and 82% in pts with AF. When focusing on the time after diagnosis of MPN (as opposed to the lifetime of a patient), the incidence of events in AF pts was higher only for BEs but not for TEs when compared to non‐AF pts (Supporting Information S1: Figure S4B,C). 10‐year BEFS was 97% in pts without AF and 89% in pts with AF. In current guidelines on the management of pts with AF in the general population, anticoagulants are recommended for pts with AF and an elevated CHA2DS2‐VASc score of ≥2 in men or ≥3 in women.^12^ Thus, it is likely that the increased incidence of cardiovascular comorbidities in our MPN pts with AF led to higher CHA2DS2‐VASc scores and to a higher rate of prophylactic ATTs, independently from the MPN management. The increased prevalence of bleeding after but rarely before the MPN diagnosis in this group of pts (Supporting Information S1: Figure 4C) may at least in part be explained by this increase in ATTs, as described earlier.^9^ Importantly, the fact that no difference in thromboembolic event‐free survival (TEFS) was observed between our MPN pts with AF vs. those without AF (Supporting Information S1: Figure 4B) suggests that management of AF pts in our cohort successfully prevented excess TEs in these pts. The definition of TEFS and bleeding event‐free survival (BEFS) is provided in the methods section of the supplemental file.

We then performed survival analysis after age‐matching of the cohort to reduce the influence of differences in the sample size and the age difference between the pts with vs without AF. The inferior survival outcomes were no longer observed in age‐matched analyses. The presence of AF did not have a significant overall influence on the OS, TEFS, or BEFS in MPN pts (Figure 1A–C). We speculate that this was due to the success of the combined use of ATTs and CRT in an aging MPN population at high risk for TEs due to coexisting AF. The difference in BEFS was no longer significant in age‐matched analysis, which may indicate that management of anticoagulation has become better tolerated during the past few years, possibly through the increased use of DOACs instead of VKAs, also shown in more recent MPN cohort analyses.^13^, ^14^

**Figure 1:**
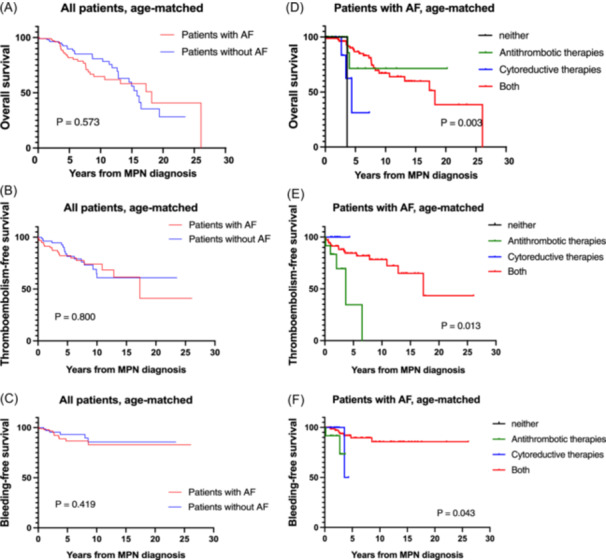
**Analyses in the age‐matched cohort. (A)** Age‐matched OS of pts with MPN (*n* = 134 each). **(B)** Age‐matched TE‐free survival (*n* = 134 each). **(C)** Age‐matched bleeding event‐free survival (*n* = 134 each). **(D)** OS stratified by antithrombotic and cytoreductive therapy in MPN pts with AF with antithrombotic or cytoreductive therapy, neither or both. Cox regression analyses: neither versus both (HR = 12.313; 95% CI 1.494–101.453, P = 0.020), antithrombotic therapies versus both (HR = 0.778, 95% CI 0.182–3.335, P = 0.736), cytoreductive therapies versus both (HR = 4.665, 95% CI 1.289–16.880, P = 0.019). **(E)** TE‐free survival stratified by antithrombotic and cytoreductive therapy in MPN pts with AF with antithrombotic or cytoreductive therapy, neither or both. Cox regression analyses: neither versus both (HR = 0.000, 95% CI 0.000–NE, P = 0.993), antithrombotic therapies versus both (HR = 4.351, 95% CI 1.465–12.926, P = 0.008), cytoreductive therapies versus both (HR = 0.000, 95% CI 0.000–NE, P = 0.982). **(F)** Bleeding‐free survival stratified by antithrombotic and cytoreductive therapy in MPN pts with AF with antithrombotic or cytoreductive therapy, neither or both. Cox regression analyses: neither versus both (HR = 0.000, 95% CI 0.000–NE, P = 0.988), antithrombotic therapies vs both (HR = 6.567, 95% CI 1.49–28.97, P = 0.013), cytoreductive therapies vs both (HR = 2.857, 95% CI 0.32–25.35, P = 0.346). NE, non‐estimable.

The importance of ATTs and CRTs was evident from our study, particularly in pts with coexisting AF. OS was worse when AF pts received neither of the therapies, or when they were treated only with CRTs (Figure 1D). Compared to patients who received both therapies, patients who received neither therapy or only CRTs had significantly inferior OS (5‐year OS for neither: not obtainable, for only CRTs: 31.3%, for Both: 86.7%). These significant differences were observed only in the AF group (*P* = 0.003). The result of the analysis of the non‐AF group is shown in Supporting Information S1: Figure S5A.

An additional OS analysis was conducted using a 1:1 matched data set with a larger number of matching factors, including age, congestive heart failure, diabetes mellitus, arterial hypertension, abnormal renal function, and a history of thromboembolic and vascular events. This analysis yielded a similar result as the matching analysis using age alone (Supporting Information S1: Figure S9).

Similarly, the worst TEFS and BEFS was observed in pts with AF who were treated only with ATT (Figure 1E,F) compared to pts treated with combined therapy with ATT and CRT.

Through age‐matched OS, TEFS, and BEFS analyses, we observed that MPN pts with AF treated with combined ATT and CRTs have better prognoses than those with ATT or CRT alone. We would like to emphasize that all age‐matched survival analyses stratified by ATT and CRT were significant only in AF patients. In non‐AF patients, OS, TEFS, and BEFS stratified by ATT and CRT showed no significance, as shown in Supporting Information S1: Figure S5. OS, TEFS, and BEFS analyses, separate for ATT and CRT, can be found in Supporting Information S1: Figures S6–S8.

Our data demonstrate the beneficial effects of MPN‐specific CRT in addition to ATT for the prevention of TEs and BEs in MPN pts with coexisting AF. As outlined in current management guidelines for MPN, the risks of thromboembolism and bleeding have to be carefully balanced.^2^, ^15^ One of the limitations of our study is that the prevalence of newly developed AF along with MPN over time could not be assessed in our study. AF was recorded at the time of registration, and it is hence unclear if the AF occurred before or after the MPN diagnosis. Moreover, while we observed a higher prevalence of AF in the MPN population compared to the non‐MPN population, due to the observational nature of our study, we cannot prove a causal relationship, but can only suggest this interesting association. Finally, whether the ATTs were discontinued after the initiation of CRT was not consistently documented in this study. In future studies, such data could enable more nuanced analyses and offer deeper insights into the interplay between AF and MPN.

However, one of the strengths of our study is the large number of pts and the inclusion of all major MPN subtypes. It adds significant data on the management of AF in MPN pts, which are urgently needed in the absence of controlled clinical trials on this topic.

In conclusion, our study provides evidence for beneficial effects of CRT as additional treatment for MPN pts with coexisting AF, including not only elderly pts older than 60 years of age but also younger pts. Prospective studies are needed to validate these findings and determine whether pts with different MPN subtypes need to be managed differently.

## AUTHOR CONTRIBUTIONS

The authors confirm their individual contributions to the manuscript as follows: Hyunyee Rosa Cho (Submitting author): Conceptualized and designed the study, collected the data, performed the statistical analyses, performed most of the data interpretation, drafted the original manuscript, and contributed to the critical review and revision of the manuscript. Steffen Koschmieder (Corresponding Author): Contributed to the collection of bioregistry data, contributed to interpretation of data evaluation, secured funding, provided supervision throughout the project, and participated in the review and revision of the manuscript. Susanne Isfort, Kim Kricheldorf, Frank Stegelmann, Martine Klausmann, Florian Heidel, Martin Griesshammer, Holger Schulz, Andreas Hochhaus, Joachim Göthert, Rudolf Schlag, Wiebke Hollburg, Lino Teichmann, Katja Sockel, Stefan Wilop, Deniz Gezer, Martin Kirschner, Konstanze Döhner, and Tim H. Brümmendorf contributed to the collection of bioregistry data, contributed to interpretation of data evaluation, and participated in the review of the manuscript. Each author reviewed the manuscript, believes it represents valid work, and approves it for submission.

## CONFLICT OF INTEREST STATEMENT

Susanne Isfort reports advisory board honoraria from GSK, Pfizer, Incyte, and Novartis, honoraria from Novartis, BMS, Pfizer, Incyte, AOP Orphan; and other financial support (e.g., travel support) from Alexion, Novartis, Pfizer, Mundipharma, Roche, Hexal, and AOP Orphan. Frank Stegelmann reports consulting fees from BMS/Celgene, Incyte, MorphoSys, Novartis and received honoraria from AbbVie, AOP Orphan, Incyte, Novartis, and Pfizer. Florian H. Heidel has received research funding from Novartis, Celgene/BMS, and CTI BioPharma and has served as a consultant for Novartis, BMS, AOP, Janssen, Abbvie, GSK, and Kartos. Martin Griesshammer received consulting fees from AOP Orphan, Novartis, BMS, AbbVie, Pfizer, Roche, Janssen, Gilead, AstraZeneca, Sierra, Lilly, GSK and reports honoraria from AOP Orphan, Novartis, BMS, AbbVie, Pfizer, Roche, Janssen, Gilead, AstraZeneca, Sierra, Lilly, and GSK. Andreas Hochhaus reports Research support by Novartis, BMS, Pfizer, Incyte, Enliven, TERNS. Lino Teichmann reports honoraria from AOP Pharma, BMS, Jazz. Pharmaceuticals, Novartis, and Sobi and consultancy for Astellas, Blueprint. Medicines, BMS, GSK, Pfizer, and Sobi. Katja Sockel received lecture fees from BMS, Novartis, GSK, AbbVie, Jazz, advisory fees from BMS, Novartis, GSK, Blueprint, and SOBI R, and reports research support from Active Biotech. Stefan Wilop reports advisory board honoraria and presentation fees from BMS. Deniz Gezer reports advisory board activity for AMGEN, Takeda and Celgene and travel money from AMGEN, Celgene, and Bristol‐Myers Squibb. Konstanze Döhner received research support from Novartis, Celgene/BMS, Astellas and Agios, received honoraria from Novartis, Janssen, Celgene/BMS, Daiichi Sankyo, JAZZ, Rosche and GSK. Reports advisory board honoraria from Novartis, Janssen, Celgene/BMS, Daiichi Sankyo, JAZZ, Rosche, AbbVie, and GSK. Tim H. Brümmendorf reports COI from Novartis, Pfiyer, Synlab, Incyte, Merck, and Rosche. Steffen Koschmieder received research funding from Geron, Janssen, AOP Pharma, and Novartis; received consulting fees from Pfizer, Incyte, Ariad, Novartis, AOP Pharma, Bristol Myers Squibb, Celgene, Geron, Janssen, CTI BioPharma, Roche, Bayer, GSK, Sierra Oncology, and PharmaEssentia; received payment or honoraria from Novartis, BMS/Celgene, and Pfizer; received travel/accommodation support from Alexion, Novartis, Bristol Myers Squibb, Incyte, AOP Pharma, CTI BioPharma, Pfizer, Celgene, Janssen, Geron, Roche, AbbVie, GSK, Sierra Oncology, and Kartos; had a patent issued for a BET inhibitor at RWTH Aachen University; participated on advisory boards for Pfizer, Incyte, Ariad, Novartis, AOP Pharma, BMS, Celgene, Geron, Janssen, CTI BioPharma, Roche, Bayer, GSK, Sierra Oncology, and PharmaEssentia, and is an Editor of *HemaSphere*. The remaining authors declare no conflict of interest.

## FUNDING

This work was supported by the German Research Foundation (DFG) within the Clinical Research Unit CRU344 to S.K. (KO 2155/9‐2, project number 417911533) and T.B. (BR 1782/5‐2 and BR 1782/6‐1, project number 428857858) and Center for Integrated Oncology, CIO‐Aachen.

## Supporting information

Supporting information.

## Data Availability

The data that support the findings of this study are available in the supplementary material of this article.
