# Supplementary material for: Mitigation of atrial fibrillation‐related complications with antithrombotic and cytoreductive therapy in patients with Myeloproliferative Neoplasms: Implications from the GSG‐MPN bioregistry
Source: Hemasphere. 2025 Mar 17;9(3):e70090. doi: 10.1002/hem3.70090 (PMC11911930; doi:10.1002/hem3.70090)
Supplement: Supplementary file 1 — Supporting information. [file HEM3-9-e70090-s001.docx]

Table of Contents

[Introduction 2](#_Toc185404897)

[Materials and Methods 3](#_Toc185404898)

[Patient Characteristics 6](#_Toc185404899)

[1) General characteristics 6](#_Toc185404900)

[2) Comparison of MPN pts with vs. without AF 8](#_Toc185404902)

[Logistic regression analyses 12](#_Toc185404903)

[1) Age-matched univariate logistic regression analysis of AF 13](#_Toc185404904)

[2) Potential risk factors for TE in MPN pts with coexisting AF 14](#_Toc185404905)

[3) Potential risk factors for bleeding events in MPN pts with coexisting AF 16](#_Toc185404906)

[4) Supplemental tables of detaled logistic regression 17](#_Toc185404907)

[5) Discussion regarding logistic regression analyses 33](#_Toc185404908)

[Supplemental Figures 35](#_Toc185404909)

# Introduction

In addition to progression to secondary myelofibrosis and leukemic transformation, vascular complications such as thromboembolic events (TE) and severe bleeding events (BE) are the major causes of morbidity and mortality of patients with MPN ^1^. Patients (pts) with MPN exhibit a hypercoagulable state, as demonstrated by elevated blood counts, activation of platelets, leukocytes, and endothelial cells, the presence of the JAK2V617F mutation, as well as increased circulating procoagulant microparticles and the occurrence of an acquired activated protein C resistance (reviewed in ^2^ and ^3^). Disease associated bleeding risk due to acquired von Willebrand syndrome, platelet dysfunction, antiplatelet agents (APA), and thrombocytopenia are known as major causes of disturbance in primary hemostasis in MPN patients ^4^. Secondary hemostasis can be disturbed due to anticoagulation therapy, acquired hemophilia ^5^, liver dysfunction or infection-induced disseminated intravascular coagulopathies ^4^.

Atrial fibrillation (AF) is the most common sustained arrhythmia worldwide, affecting up to 2% of the general population, with increasing prevalence with age ^6, 7^. AF predisposes to TE contributing to increased morbidity and mortality. Ischemic stroke is the predominant complication, but other TE such as deep vein thrombosis and pulmonary embolism are also increased in patients with AF ^8, 9^.

The incidence of AF has rarely been studied in MPN, but was suggested to be higher compared to the general population in one retrospective report of 96 MPN pts with AF vs. 617 MPN pts without AF ^10^. The same study found that MPN pts with AF had a higher frequency of cardiovascular risk factors (CVRF) and thrombosis and a shorter thrombosis-free survival than those pts without AF ^10^. However, in a separate retrospective study, comparing 63 pts with polycythemia vera (PV) and AF to 124 control pts with AF only, all of whom were on anticoagulant treatment, no increased incidence of thrombosis was found ^11^. Both studies found no increase in major bleeding events. However, none of the two studies reported the use and effects of cytoreductive therapy in their patient cohorts.

Therefore, we conducted this retrospective analysis of 2,780 MPN pts enrolled in the GSG-MPN bioregistry, in order to identify the characteristics of AF in MPN patients, to assess the AF-associated risk factors for TE and severe bleeding events in MPN patients and to assess the benefit-risk profile of antithrombotic therapies (ATTs) alone or in combination with MPN-specific cytoreductive therapies.

# Materials and Methods

German Study Group MPN bioregistry (GSG-MPN bioregistry) is an ambispective observational study of MPN patients (pts) with over 70 centers participating, including university hospitals, community hospitals, and office-based hematologist/oncologists. Pts with a confirmed MPN diagnosis according to the WHO classification of 2008 ^12^ or 2016 ^13^ or IWG-MRT criteria, who were 18 years of age or older were included in the bioregistry after informed consent was obtained. Pts deemed unable to provide written informed consent due to neurological or psychological impairment and pts who did not agree to registration were not included in the bioregistry. Pts are being prospectively followed after enrollment into the registry, annually recording both their clinical data and collecting biomaterial. In addition, retrospective data on their disease course and medical history are being recorded as well, resulting in an ambispective bioregistry. The bioregistry was approved by the Ethics Committee of the Medical Faculty of RWTH Aachen University (EK 127/12) as well as by each local Ethics Committees of the participating medical centers. Recruitment started in August 2012, and the present analysis is based on the data of 2,780 pts from the first registration and annual follow up up to 3 years, with a data cut-off date of January 2020.

The data were analyzed by SPSS version 27 for Mac (SPSS Inc., Chicago, IL, USA). Mann-Whitney and Fisher’s exact tests were used for comparing the pts group with vs without AF. The information for AF was collected at the time of registration. P values <0.05 were considered significant, and p values <0.09 were considered as a trend. Logistic regression was used to detect predictors of thromboembolic/bleeding events. To observe the effect of thromboembolic/vascular events (TE) history on TE recurrence, we performed additional analyses, where only those TE´s which occurred at or after the MPN diagnosis were collected and were set as the dependent factor. Kaplan-Meier survival analysis and Cox proportional hazard modeling was used to analyze OS, TE-free survival (TEFS) and bleeding event-free survival (BEFS). TEFS in our study is defined as the duration of survival from the date of MPN diagnosis until the occurrence of a thromboembolic/vascular event, measured in years. TE in our data included the following events: deep vein thrombosis (DVT), sinus vein thrombosis, thrombophlebitis, Budd-Chiari syndrome, pulmonary embolism, coronary artery disease/myocardial infarction (CAD/MI), cerebral ischemia, and peripheral artery occlusion.

Meanwhile, BEFS is defined as the period from the date of MPN diagnosis until a BE occurs, also measured in years. A BE was documented in our registry when patients required medical treatment due to bleeding. The following events were included: upper and lower gastrointestinal hemorrhage, intracranial hemorrhage, abdominal bleeding (e.g., from the spleen or liver), and urogenital tract bleeding. Bleeding events such as epistaxis, soft tissue bleeding, posttraumatic and postoperative bleedings were also documented as bleeding events.

We analyzed MPN subtypes PV, essential thrombocythemia (ET), primary myelofibrosis (PMF), MPN unclassifiable (MPN-U), post-PV-myelofibrosis (postPV-MF) and post-ET myelofibrosis (post-ET-MF) separately. Other MPNs were subcategorized as “Others”. For the logistic regression model, we categorized the laboratory parameters into groups according to normal ranges to analyze the effect of abnormal laboratory values.

We calculated the risk factors based on the CHA2DS2-VASc and HAS-BLED scoring systems ^14, 15^. CHA2DS2-VASc score is a risk stratification method that can be used to assess the risk of TE in pts with AF. This system counts congestive heart failure, hypertension, age ≥ 75 years (two points), diabetes mellitus, prior stroke or transient ischemic attack or thromboembolism (two points), vascular disease, age 65 to 74 years and sex category. The age of pts included in our analysis is from the first registration, not at first diagnosis, since other variables were also used from the time of first registration. Use of HAS-BLED score is recommended to assess the 1-year risk of major bleeding in AF pts ^7, 15, 16^. Criteria include uncontrolled arterial hypertension, abnormal renal function abnormal liver function, prior history of stroke, prior major bleeding or predisposition to bleeding, labile international normalized ratio (INR), age > 65 years, prior alcohol or drug usage history and medication usage predisposing to bleeding.

Since age is known to be a risk factor for TE in both normal population^17^ and in MPN pts^18, 19^, we performed an age-matched analysis. 134 pts with AF were randomly age-matched with non-AF patient group via SPSS software.

# Patient Characteristics

### General characteristics

Supplemental Table S1: General characteristics (n=2,780)

| **Variables** |  | | **N** | **%** | **Median (IQR)** |
| --- | --- | --- | --- | --- | --- |
| Age at first registration  (n=2,780) | < 65 | | 1488 | 53.5 | 63.8 (52-74) |
|  | 65- 74 | | 640 | 23.0 |  |
|  | > 74 | | 652 | 23.5 |  |
| Sex  (n=2,780) | Men | | 1370 | 49.3 |  |
|  | Women | | 1410 | 50.7 |  |
| MPN Subtypes  (n=2,731) | Polycythemia vera | | 829 | 30.4 |  |
|  | Essential Thrombocythemia | | 940 | 34.4 |  |
|  | Primary myelofibrosis | | 599 | 21.9 |  |
|  | PostPV- myelofibrosis | | 73 | 2.7 |  |
|  | PostET- myelofibrosis | | 82 | 3.0 |  |
|  | MPN-Unclassifiable | | 146 | 5.3 |  |
|  | Others | | 62 | 2.3 |  |
| Leucocyte count*  (n=2,659) | < 3.5/nl | | 81 | 3.0 | 9.1 (7-12) |
|  | 3.5-10/nl | | 1495 | 56.2 |  |
|  | > 10/nl | | 1083 | 40.8 |  |
| Hematocrit*  (n=2,613) | ≤ 45/nl | | 1833 | 70.1 | 42.0 (37-46) |
|  | > 45/nl | | 780 | 29.9 |  |
| Platelet count *  (n=2,641) | < 150/nl | | 245 | 9.3 | 485.0 (288-727) |
|  | 150-450/nl | | 958 | 36.3 |  |
|  | > 450/nl | | 1438 | 54.4 |  |
| LDH*  (n=2,372) | ≤ 250U/L | | 947 | 39.9 | 278.0 (220-403) |
|  | > 250U/L | | 1425 | 60.1 |  |
| Splenomegaly  (n=2,494) | Yes | | 1380 | 55.3 |  |
|  | No | | 1114 | 44.7 |  |
| Atrial fibrillation  (n=2,780) | Yes | | 134 | 4.8 |  |
|  | No** | | 2646 | 95.2 |  |
| Congestive heart failure  (n=2,395) | Yes | | 177 | 7.4 |  |
|  | No** | | 2218 | 92.6 |  |
| Myocardial Infarction  (n=2,407) | Yes | | 145 | 6.0 |  |
|  | No** | | 2262 | 94.0 |  |
| Diabetes mellitus  (n=2,780) | Yes | | 230 | 8.3 |  |
|  | No** | | 2550 | 91.7 |  |
| Hypertension  (n=2,415) | Yes | | 1191 | 49.3 |  |
|  | No | | 1224 | 50.7 |  |
| CHA2DS2-VASc  (n=2,780) | M ≤ 1, F ≤ 2 | | 1553 | 55.9 | 2.0 (1-3) |
|  | M > 1, F > 2 | | 1227 | 44.1 |  |
| HASBLED  (n=2,780) | ≤ 2 | | 2201 | 79.2 | 2.0 (1-2) |
|  | > 2 | | 579 | 20.8 |  |
| Antithrombotic therapy  (n=2,780) | Antiplatelet therapy | | 1593 | 57.3+ |  |
|  | Vitamin K antagonist | | 198 | 7.1+ |  |
|  | DOAC | | 179 | 6.4+ |  |
|  | Heparin | | 78 | 2.8+ |  |
|  | Antiplatelet and Anticoagulation*** | | 1867 | 67.2+ |  |
| MPN therapy  (n=2,780) | Anagrelide | | 451 | 16.2+ |  |
|  | Hydroxyurea (HU) | | 1444 | 51.9+ |  |
|  | Ruxolitinib (RUX) | | 768 | 27.6+ |  |
|  | Interferon (IFN) | | 312 | 11.2+ |  |
|  | Imide | | 32 | 1.2+ |  |
|  | MPN Therapy*** | | 2023 | 72.8 |  |
| Driver mutations  (n=2,284) | Yes | | 2146 | 93.8 |  |
|  | No | | 141 | 6.2 |  |
|  | JAK2  (n=2508) | Yes | 1842 | 73.4 |  |
|  |  | No | 666 | 26.6 |  |
|  | CalR  (n=1175) | Yes | 273 | 23.2 |  |
|  |  | No | 902 | 76.8 |  |
|  | MPL  (n=1099) | Yes | 59 | 5.4 |  |
|  |  | No | 1099 | 94.6 |  |
| Thromboembolic event  (n=2,601) | Yes | | 979 | 37.6 |  |
|  | No | | 1622 | 62.4 |  |
| Bleeding  (n=2,537) | Yes | | 135 | 5.3 |  |
|  | No | | 2402 | 94.7 |  |

*: results at first registration

** no or not documented

*** one or more than one therapy

+:percentage from total 2,780

Supplemental Table S1 lists the main patient characteristics of all 2,780 pts. There were 1,370 male and 1,410 female pts, with a median age of 63.8 years. Among these pts, 979 were documented with TE and 135 pts were documented with BE. Supplemental Figure S1 shows the increase in AF prevalence with age and supplemntal Figure S2 shows the age distribution among MPN pts with AF and without AF. The presence of splenomegaly and relevant cardiovascular risk factors are shown in supplemental Table S1. Table S1 also demonstrates the MPN-specific cytoreductive therapies and driver mutation analysis.

Supplemental Table S2: Antithrombotic therapy in all patients (n=2,780)

|  | with antithrombotic therapy (%row) | without antithrombotic therapy (%row) | P |  |
| --- | --- | --- | --- | --- |
| CHA2DS2-VASc score |  |  | <0.001 | ^+*^ |
| M ≤ 1, F ≤ 2 | 952 (61.3) | 601 (38.7) |  |  |
| M > 1, F > 2 | 915 (74.6) | 312 (25.4) |  |  |
| HASBLED Score |  |  | <0.001 | ^+*^ |
| ≤ 2 | 1312 (59.6) | 889 (40.4) |  |  |
| > 2 | 555 (95.8) | 24 (4.2) |  |  |

+ Fisher’s Exact test for categorized variables

In this study, almost half of the pts showed high CHA2DS2-VASc scores (for men higher than 1, for women higher than 2), suggesting that they had an indication for anticoagulation therapy (Supplemental Table S2). Approximately 1/5 of the pts had HASBLED scores higher than 2, which is a relative contraindication for anticoagulation in pts with AF ^20^. The majority of the pts were treated with one or more ATTs.

### Comparison of MPN pts with vs. without AF

Supplemental Table S3: Comparisons of MPN pts with (n=134) and without (n=2,646) atrial fibrillation (not age-matched)

|  |  | | With AF^a^ (n=134) | | | Without AF^a^ (n=2,646) | | | |  |
| --- | --- | --- | --- | --- | --- | --- | --- | --- | --- | --- |
| Variables |  | | N | % column | Median  (IQR) | N | % column | Median  (IQR) | P |  |
| Age  (n=2780) | <65 | | 18 | 13.4 | 76.0  (70-81) | 1,470 | 55.6 | 63.1  (52-73) | **<0.001**  **<0.001** | ^*^  ^+*^ |
|  | 65- 74 | | 36 | 26.9 |  | 604 | 22.8 |  |  |  |
|  | >74 | | 80 | 60.3 |  | 572 | 21.6 |  |  |  |
| Sex  (n=2780) | Men | | 74 | 55.2 |  | 1,296 | 49.0 |  | 0.184 | ^+^ |
|  | Women | | 60 | 44.8 |  | 1,350 | 51.0 |  |  |  |
| MPN Subtype^b^  (n=2731) | PV | | 42 | 31.3 |  | 787 | 30.3 |  | 0.278 | ^+^ |
|  | ET | | 41 | 30.6 |  | 899 | 34.6 |  |  | ^+^ |
|  | PMF | | 27 | 20.1 |  | 572 | 22.0 |  |  | ^+^ |
|  | PostPV-MF | | 6 | 4.5 |  | 67 | 2.6 |  |  | ^+^ |
|  | PostET-MF | | 2 | 1.5 |  | 80 | 3.1 |  |  | ^+^ |
|  | MPN- U | | 11 | 8.2 |  | 135 | 5.2 |  |  | ^+^ |
|  | Others | | 5 | 3.7 |  | 57 | 2.2 |  |  | ^+^ |
| Leucocyte count  (n=2,659) | < 3.5/nl | | 2 | 1.5 | 9.4  (8-14) | 79 | 3.1 | 9.0  (7-12) | **0.024**  0.574 | ^*^  ^+^ |
|  | 3.5-10/nl | | 74 | 55.2 |  | 1,421 | 56.3 |  |  |  |
|  | > 10/nl | | 58 | 43.3 |  | 1,025 | 40.6 |  |  |  |
| Hematocrit  (n=2,613) | ≤ 45/nl | | 80 | 60.6 | 42.5  (36-48) | 1,753 | 70.7 | 42.0  (37-46) | 0.340  **0.019** | ^+*^ |
|  | > 45/nl | | 52 | 39.4 |  | 728 | 29.3 |  |  |  |
| Platelet count  (n=2,641) | < 150/nl | | 11 | 8.2 | 451.0  (298-697) | 234 | 9.3 | 488.0  (287-729) | 0.385  0.505 | ^+^ |
|  | 150-450/nl | | 55 | 41.0 |  | 903 | 36.0 |  |  |  |
|  | > 450/nl | | 68 | 50.7 |  | 1,370 | 54.6 |  |  |  |
| LDH^c^  (n=2,372) | ≤ 250U/L | | 43 | 35.0 | 311.0  (232-403) | 904 | 40.2 | 277.0  (218-402) | 0.099  0.258 | ^+^ |
|  | > 250U/L | | 80 | 65.0 |  | 1345 | 59.8 |  |  |  |
| Myocardial infarction  (n=2,407) | Yes | | 10 | 7.9 |  | 135 | 5.9 |  | 0.339 | ^+^ |
|  | No^**^ | | 117 | 92.1 |  | 2,145 | 94.1 |  |  |  |
| Congestive heart failure  (n=2,395) | Yes | | 28 | 21.9 |  | 149 | 6.6 |  | **<0.001** | ^+*^ |
|  | No^**^ | | 100 | 78.1 |  | 2,118 | 93.4 |  |  |  |
| Diabetes mellitus  (n=2,780) | Yes | | 28 | 20.9 |  | 202 | 7.6 |  | **<0.001** | ^+*^ |
|  | No^**^ | | 106 | 79.1 |  | 2,444 | 92.4 |  |  |  |
| Hypertension  (n=2,415) | Yes | | 90 | 72.6 |  | 1,101 | 48.1 |  | **<0.001** | ^+*^ |
|  | No^**^ | | 34 | 27.4 |  | 1,190 | 51.9 |  |  |  |
| Splenomegaly  (n=2,494) | Yes | | 63 | 52.5 |  | 1,317 | 55.5 |  | 0.573 | ^+^ |
|  | No^**^ | | 57 | 47.5 |  | 1,057 | 44.5 |  |  |  |
| CHA2DS2-VASc  (n=2,780) | M ≤ 1, F ≤ 2 | | 17 | 12.7 | 3.0  (2-4) | 1,536 | 58.0 | 2.0  (1-3) | **<0.001**  **<0.001** | ^*^  ^+*^ |
|  | M > 1, F > 2 | | 117 | 87.3 |  | 1,110 | 42.0 |  |  |  |
| HAS-BLED  (n=2,780) | ≤ 2 | | 74 | 55.2 | 2.0  (2-3) | 2,127 | 80.4 | 2.0  (1-2) | **<0.001**  **<0.001** | ^*^  ^+*^ |
|  | > 2 | | 60 | 44.8 |  | 519 | 19.6 |  |  |  |
| TE^d^ at or after diagnosis  (n=2780) | Yes | | 21 | 15.7 |  | 354 | 13.4 |  | 0.437 | ^+^ |
|  | No^**^ | | 113 | 84.3 |  | 2,292 | 86.6 |  |  |  |
| TE^d^ regardless of time  (n=2,601) | Yes | | 67 | 51.5 |  | 912 | 36.9 |  | **0.001** | ^+*^ |
|  | No^**^ | | 63 | 48.5 |  | 1,559 | 63.1 |  |  |  |
| Types of TE^d^  (n=977) | Arterial | | 46 | 68.7 |  | 488 | 53.6 |  | **0.025** | ^+*^ |
|  | Venous | | 13 | 19.4 |  | 317 | 34.8 |  |  |  |
|  | Both | | 8 | 11.9 |  | 105 | 11.5 |  |  |  |
| Bleeding regardless of time  (n=2,537) | Yes | | 13 | 10.2 |  | 122 | 5.1 |  | **0.022** | ^+*^ |
|  | No^**^ | | 114 | 89.8 |  | 2,288 | 94.9 |  |  |  |
| Bleeding event at or after diagnosis (n=2,780) | Yes | | 11 | 8.2 |  | 83 | 3.1 |  | **0.005** | ^+*^ |
|  | No^**^ | | 123 | 91.8 |  | 2,563 | 96.9 |  |  |  |
| Antithrombotic therapy^e^  (n=2,780) | Antiplatelet therapy | Yes | 51 | 38.1 |  | 1,542 | 58.3 |  | **<0.001** | ^+*^ |
|  |  | No | 83 | 61.9 |  | 1,104 | 41.7 |  |  |  |
|  | VKA | Yes | 47 | 35.1 |  | 151 | 5.7 |  | **<0.001** | ^+*^ |
|  |  | No | 87 | 64.9 |  | 2,495 | 94.3 |  |  |  |
|  | DOAC | Yes | 50 | 37.3 |  | 129 | 4.9 |  | **<0.001** | ^+*^ |
|  |  | No | 84 | 62.7 |  | 2,517 | 95.1 |  |  |  |
|  | Heparin | Yes | 6 | 4.5 |  | 72 | 2.7 |  | 0.273 | ^+^ |
|  |  | No | 128 | 95.5 |  | 2,574 | 97.3 |  |  |  |
|  | Any type of antithrombotic^***^ | Yes | 115 | 85.8 |  | 1752 | 66.2 |  | **<0.001** | ^+*^ |
|  |  | No | 19 | 14.2 |  | 894 | 33.8 |  |  |  |
| MPN Therapy  (n=2,780) | Anagrelide | Yes | 23 | 17.2 |  | 428 | 16.2 |  | 0.720 | ^+^ |
|  |  | No^**^ | 111 | 82.8 |  | 2,218 | 83.8 |  |  |  |
|  | HU | Yes | 86 | 64.2 |  | 1,358 | 51.3 |  | **0.004** | ^+*^ |
|  |  | No | 48 | 35.8 |  | 1,288 | 48.7 |  |  |  |
|  | Ruxolitinib | Yes | 35 | 26.1 |  | 733 | 27.7 |  | 0.767 | ^+^ |
|  |  | No | 99 | 73.9 |  | 1,913 | 72.3 |  |  |  |
|  | Interferon | Yes | 13 | 9.7 |  | 299 | 11.3 |  | 0.674 | ^+^ |
|  |  | No | 121 | 90.3 |  | 2,347 | 88.7 |  |  |  |
|  | Imide | Yes | 3 | 2.2 |  | 29 | 1.1 |  | 0.198 | ^+^ |
|  |  | No | 131 | 97.8 |  | 2,617 | 98.9 |  |  |  |
|  | Any type of MPN Therapy^***^ | Yes | 102 | 76.1 |  | 1921 | 72.6 |  | 0.426 | ^+^ |
|  |  | No | 32 | 23.9 |  | 725 | 27.4 |  |  |  |
| Driver Mutations | JAK2  (n=2,508) | Yes | 94 | 74.6 |  | 1,748 | 73.4 |  | 0.836 | ^+^ |
|  |  | No | 32 | 25.4 |  | 634 | 26.6 |  |  |  |
|  | CalR  (n=1,175) | Yes | 9 | 18.8 |  | 264 | 23.4 |  | 0.600 | ^+^ |
|  |  | No | 39 | 81.3 |  | 863 | 76.6 |  |  |  |
|  | MPL  (n=1,099) | Yes | 6 | 12.2 |  | 53 | 5.0 |  | **0.042** | ^+*^ |
|  |  | No | 43 | 87.8 |  | 997 | 95.0 |  |  |  |

a. AF: atrial fibrillation. History of successful cardioversion before the date of MPN diagnosis were not considered to have AF. b. polycythemia vera (PV), essential thrombocythemia (ET), primary myelofibrosis (PMF), MPN unclassifiable (MPN-U), postPV-myelofibrosis (postPV-MF), post-ET myelofibrosis (postET-MF) c. LDH: lactate dehydrogenase. d: TE: thromboembolic events. e. vitamin K antagonist (VKA), direct oral anticoagulation (DOAC)

+: Fisher’s Exact test for categorized variables

*: P<0.05

**: No or not documented

***: any type of therapy, these patients could have been treated with several therapies, either simultaneously or separately

We compared the variables between the pts with vs without AF, as shown in Supplemental Table S3. Among the 2,780 pts, AF was documented in 134 pts. Pts with AF were older than non-AF pts, they had significantly more comorbidities and CVRF, and they had a higher incidence of MPL mutations than those without AF (Supplemental Table S3). CHA2DS2-VASc scores were significantly higher in median value and higher percentage of pts had increased CHA2DS2-VASc score (M>1, F>2), and while the median of HAS-BLED score was not different between AF and non-AF pts, the percentage of pts with HAS-BLED score > 2 was higher in the patient group with AF. The incidence of both TE and BE occurring during lifetime of a patient was observed to be higher in the AF group.

As observed in the general population ^10^, the median age in our MPN cohort was higher in pts with AF than those without AF. The median age of pts with AF was comparable with that of the normal population with AF without MPN (about 75 years) ^21^. The prevalence of AF in pts above the age of 80 years in our MPN cohort was somewhat higher than in the general population (18.2% [Supplemental Fig. S2] vs. 8.8% in the FRAMINGHAM study ^22^). One potential limitation of our study is the use of the age at registration instead of at first MPN diagnosis. However this was done for consistency, because all of the other variables were taken from first registration. Also, age at registration may better reflect the current situation of a patient with a chronic malignancy whose diagnosis may have been established years ago.

The percentage of pts with total documented TE in MPN pts, primarily arterial TE, was significantly higher in the group with AF. However, the occurrence of TE at or after the diagnosis of MPN was similar between the two groups. This suggests that TE occurred throughout the patients' lives and were at least partially related to AF. Supplemental Figure S3A shows the number of TE over time with relation to the date of diagnosis. The higher percentage of arterial TE in AF pts vs. non-AF pts, as seen in the entire MPN cohort, was no longer observed after age-matching, suggesting that all pts (MPN pts as well as non-MPN pts) develop a preponderance of arterial over venous TE with increasing age, as has been described before ^1, 23, 24^.

The total incidence of BE and BE occurring at or after MPN diagnosis was significantly higher in the AF group. As seen in Supplemental Figure S3B, most of the bleeding occurred after the MPN diagnosis, as described previously^23^. Cardiovascular comorbidities are reported to be risk factors for the development of AF ^22, 25, 26^. In our cohort, MPN pts with AF had significantly higher rates of CVRF compared to MPN pts without AF. In current guidelines on the management of pts with AF in the general population, anticoagulants are recommended for pts with AF and an elevated CHA2DS2-VASc score of ≥ 2 in men or ≥ 3 in women ^16^. Thus, it is possible, if not, likely that the increased incidence of cardiovascular comorbidities in our MPN pts with AF led to higher CHA2DS2-VASc scores and to a higher rate of prophylactic ATTs, independently from the MPN management. The increased prevalence of bleeding after but rarely before the MPN diagnosis in this group of pts may at least in part be explained by this increase in ATTs, as described earlier ^23^.

In our MPN cohort, approx. 70% of the pts were treated with ATT, and this fraction was higher in pts with AF than without AF. While APA use was more frequent in pts without AF, vitamin K antagonist (VKA) and direct oral anticoagulant (DOAC) use was more frequent in AF pts. The differences suggest that combined treatment of APA and anticoagulants was rare, as reported previously ^27^. The percentage of pts treated with one or more MPN specific cytoreductive therapies was not significantly higher in the patient group with AF, but the proportion of pts who were treated with hydroxycarbamide (HU) was higher in pts with AF (Supplemental Table S3).

However, importantly, the fact that no difference in TEFS was observed between our MPN pts with AF vs. those without AF (Figure 1B) suggests that management of AF pts in our cohort successfully prevented excess TE in these pts.

# Logistic regression analyses

Logistic regression analyses were performed to assess the AF-associated risk factors for TE and severe BE in MPN pts. We calculated the risk factors based on the CHA2DS2-VASc and HAS-BLED scoring systems ^14, 15^. CHA2DS2-VASc score is a risk stratification method that can be used to assess the risk of TE in pts with AF. This system counts congestive heart failure, hypertension, age ≥ 75 years (two points), diabetes mellitus, prior stroke or transient ischemic attack or thromboembolism (two points), vascular disease, age 65 to 74 years and sex category. The age of pts included in our analysis is from the first registration, not at first diagnosis, since other variables were also used from the time of first registration. Use of HAS-BLED score is recommended to assess the 1-year risk of major bleeding in AF pts ^7, 15, 16^. Criteria include uncontrolled arterial hypertension, abnormal renal function, abnormal liver function, prior history of stroke, prior major bleeding or predisposition to bleeding, labile international normalized ratio (INR), age > 65 years, prior alcohol or drug usage history and medication usage predisposing to bleeding.

### Age-matched univariate logistic regression analysis of AF

Supplemental Table S4: Univariate logistic regression for thromboembolic and bleeding events (age-matched, total n=238)

| Risk factor | OR | 95% CI | P |
| --- | --- | --- | --- |
| **Age-matched** |  |  |  |
| TE^a^ before diagnosis of MPN |  |  |  |
| Atrial fibrillation Y vs N | 1.364 | 0.789 – 2.359 | 0.267 |
| TE^a^ at or after diagnosis of MPN |  |  |  |
| Atrial fibrillation Y vs N | 1.279 | 0.642 – 2.549 | 0.484 |
| BE^b^ before diagnosis of MPN |  |  |  |
| Atrial fibrillation Y vs N | n.a. | n.a. | n.a. |
| BE^b^ at or after diagnosis of MPN |  |  |  |
| Atrial fibrillation Y vs N | 1.908 | 0.685 – 5.318 | 0.217 |

a:Thromboembolic events, b: Bleeding events

As shown in our age-matched logistic regression analysis, AF did not increase the odds for TE and BE in MPN pts of our cohort (Supplemental Table S4).

### Potential risk factors for TE in MPN pts with coexisting AF

Supplemental Table S5: Variables with significance in univariate/multivariate logistic regression for thromboembolism (age-matched)

|  | Univariate logistic regression | OR | P | Multivariate logistic regression | OR | P |
| --- | --- | --- | --- | --- | --- | --- |
| **Pts with AF** |  |  |  |  |  |  |
| Thromboembolism before diagnosis of MPN  (n=39) | Leukocytosis > 10/nl vs 3.5-10/nl  Myocardial infarction Y vs N  Splenomegaly^a^ Y vs N  MPN therapy Y vs N  HU Y vs N  IFN Y vs N | 0.45  11.6  0.46  3.71  2.82  3.26 | 0.051^#^  0.003  0.054^#^  0.022  0.021  0.047 | Myocardial infarction Y vs N  MPN Therapy Y vs N | 10.90  3.59 | 0.005  0.032 |
| Thromboembolism at/after diagnosis of MPN  (n=16) | Leukocytosis > 10/nl vs 3.5-10/nl  Hematocrit > 45/nl vs ≤ 45/nl  HASBLED > 2 vs ≤ 2  Antiplatelet therapy Y vs N  Anagrelide Y vs N | 0.34  2.70  2.91  3.21  3.03 | 0.050^#^  0.046  0.033  0.018  0.038 | HASBLED > 2 vs ≤ 2  Antiplatelet therapy Y vs N | 3.35  3.65 | 0.020  0.011 |
| **Pts without AF** |  |  |  |  |  |  |
| Thromboembolism before Diagnosis of MPN  (n=31) | Age > 74 vs < 65  Hematocrit > 45/nl vs ≤ 45/nl  Myocardial infarction Y vs N  HASBLED > 2 vs ≤ 2  Antithrombotic therapy Y vs N  Anagrelide Y vs N | 6.56  2.33  20.13  4.83  2.915  0.138 | 0.076^#^  0.049  <0.001  0.001  0.043  0.059^#^ | Myocardial infarction Y vs N  HASBLED > 2 vs ≤ 2 | 21.99  4.921 | <0.001  0.003 |
| Thromboembolism at/after Diagnosis of MPN  (n=15) | HASBLED > 2 vs ≤ 2  Antiplatelet therapy Y vs N  Anagrelide Y vs N | 2.913  3.207  3.031 | 0.033  0.018  0.38 | Anagrelide Y vs N | 3.709 | 0.023 |

a. Splenomegaly was defined in accordance with Poulin et al ^28^: the longest dimension of the spleen in ultrasound longer than 11cm, or when the spleen was palpably enlarged in physical exams.

#: Borderline significance P < 0.09

In the entire population of 2,780 MPN pts of our analysis, 979 pts were documented to have more than one TE at any date during their lifetime. Among these pts, TE occurrence at or after MPN diagnosis was documented in 375 (13.5%) pts. When analyzing the multivariate risk factors for TE separately for AF vs. non-AF pts (Supplemental Table S5), we observed that, in pts with AF, increased HAS-BLED score and treatment with APA showed significantly elevated ORs for TE at/after the diagnosis of MPN. In non-AF pts (n=134) significantly increased ORs for TE at/after the MPN diagnosis were seen, when pts were treated with anagrelide. For details of each logistic regression, see Supplemental Tables S7-S11).

One of the limitations of our study is that the time between the first administration of therapies and TE was not systematically documented in the bioregistry, and this should therefore be taken into account when interpreting the results. The elevated OR for TE in antithrombotic/cytoreductive therapies should not be considered as risk factors, but rather as strong correlative factors between TE and the indication of therapies.

### Potential risk factors for bleeding events in MPN pts with coexisting AF

Supplemental Table S6: Variables with significance in univariate/multivariate logistic regression for bleeding (age-matched)

|  | Univariate logistic regression | OR | P | Multivariate logistic regression | OR | P |
| --- | --- | --- | --- | --- | --- | --- |
| Pts with AF |  |  |  |  |  |  |
| Bleeding before diagnosis of MPN (n=0) | Could not be performed since case=0 |  |  |  |  |  |
| Bleeding at/after diagnosis of MPN (n=11) | PostPV-MF Y vs N  VKA Y vs N | 6.61  5.74 | 0.043  0.013 | PostPV-MF Y vs N  VKA Y vs N | 8.82  6.46 | 0.036  0.011 |
| Pts without AF |  |  |  |  |  |  |
| Bleeding before Diagnosis of MPN (n=0) | Could not be performed since case=0 |  |  |  |  |  |
| Bleeding at/after Diagnosis of MPN (n=6) | VAK Y vs N | 7.50 | 0.032 |  |  |  |

Supplemental Figure S3B depicts the incidence of major BE over time in respect to the time point of first MPN diagnosis, showing that most BE occurred after the diagnosis of the MPN. 6 shows the logistic regression data analyses of risk factors for major BE separately for AF and non-AF pts (for details of each logistic regression, see Supplemental Tables S12-S14). Analysis for BE occurring before MPN diagnosis could not be performed due to the paucity of events. In pts with AF, a diagnosis of post-PV-MF or administration of VKA were associated with an increased OR for BE at/after MPN diagnosis. No BE occurring before the MPN diagnosis was documented among AF pts (Supplemental Table S6). In pts without AF, use of VKA was shown to be a potential risk factor for BE that occurred after the diagnosis of MPN (Supplemental Table S6).

### Supplemental tables of detaled logistic regression

Supplemental Table S7: Logistic regression for thromboembolic/vascular events before diagnosis in MPN patients with atrial fibrillation (n=134)

| **Risk factor** | | **OR^a^** | **95% CI^b^** | ***P*** |  |
| --- | --- | --- | --- | --- | --- |
| *Univariate regression* | |  |  |  |  |
| Age at first registration | 65-74 vs < 65 | 0.769 | 0.227 – 2.610 | 0.674 |  |
|  | > 74 vs < 65 | 0.807 | 0.271 – 2.408 | 0.701 |  |
| Gender Female vs male | | 1.082 | 0.512 – 2.286 | 0.837 |  |
| MPN Diagnosis | PV Yes vs No | 0.564 | 0.239 – 1.327 | 0.189 |  |
|  | ET Yes vs No | 1.410 | 0.639 – 3.113 | 0.395 |  |
|  | PMF Yes vs No | 0.820 | 0.316 – 2.132 | 0.684 |  |
|  | PostPV-MF Yes vs No | 0.474 | 0.054 – 4.191 | 0.502 |  |
|  | PostET-MF Yes vs No | 4147840812.725 | 0.000 – . | >0.999^c^ |  |
|  | MPN- Unclassifiable Yes vs No | 0.906 | 0.227 – 3.612 | 0.889 |  |
|  | Others Yes vs No | 3.875 | 0.622 – 24.158 | 0.147 |  |
| Leukocyte count | < 3.5/nl vs 3.5-10/nl | 0.000 ^c^ | 0.000 – . | >0.999^c^ |  |
|  | > 10/nl vs 3.5-10/nl | **0.454** | **0.206 – 1.003** | **0.051** | **#** |
| Hematocrit > 45/nl vs ≤ 45/nl | | 0.946 | 0.439 – 2.037 | 0.887 |  |
| Platelet count | < 150/nl vs 150-450/nl | 1.098 | 0.255 – 4.724 | 0.900 |  |
|  | > 450/nl vs 150-450/nl | 1.401 | 0.635 – 3.090 | 0.404 |  |
| LDH > 250U/L vs ≤ 250U/L | | 0.708 | 0.319 – 1.570 | 0.395 |  |
| Myocardial infarction Yes vs No | | **11.600** | **2.332 – 57.691** | **0.003** | ***** |
| Congestive heart failure Yes vs No | | 1.360 | 0.561 – 3.297 | 0.496 |  |
| Diabetes mellitus Yes vs No | | 0.968 | 0.385 – 2.430 | 0.944 |  |
| Arterial hypertension Yes vs No | | 0.896 | 0.384 – 2.093 | 0.800 |  |
| Splenomegaly Yes vs No | | **0.462** | **0.211 – 1.013** | **0.054** | **#** |
| CHA2DS2-VASc score M>1, F>2 vs M≤1, F≤2 | | 3.469 | 0.754 – 15.955 | 0.110 |  |
| Abnormal liver function Yes vs No | | 1.667 | 0.267 – 10.395 | 0.584 |  |
| Abnormal renal function | | 1.313 | 0.409 – 4.216 | 0.647 |  |
| HASBLED score >2 vs ≤2 | | 1.675 | 0.791 – 3.549 | 0.178 |  |
| Antithrombotic therapy | Yes vs No | 1.641 | 0.508 – 5.298 | 0.408 |  |
|  | Antiplatelet Yes vs No | 0.878 | 0.405 – 1.902 | 0.741 |  |
|  | Vit.K Antagonist Yes vs No | 1.230 | 0.568 – 2.665 | 0.599 |  |
|  | DOAC Yes vs No | 1.452 | 0.678 – 3.107 | 0.337 |  |
|  | Heparin No vs Yes | 0.474 | 0.054 – 4.191 | 0.502 |  |
| Driver Mutations | Yes vs No | 0.545 | 0.143 – 2.077 | 0.374 |  |
|  | JAK2 Yes vs No | 0.530 | 0.228 – 1.228 | 0.138 |  |
|  | CalR Yes vs No | 2.036 | 0.460 – 9.020 | 0.349 |  |
|  | MPL Yes vs No | 0.462 | 0.049 – 4.351 | 0.499 |  |
| MPN Therapy | Yes vs No | **3.706** | **1.206 – 11.390** | **0.022** | ***** |
|  | Anagrelide Yes vs No | 1.376 | 0.531 – 3.570 | 0.511 |  |
|  | HU Yes vs No | **2.818** | **1.172 – 6.777** | **0.021** | ***** |
|  | RUX Yes vs No | 1.162 | 0.503 – 2.684 | 0.725 |  |
|  | IFN Yes vs No | **3.245** | **1.014 – 10.380** | **0.047** | * |
|  | Imide Yes vs No | 0.000^c^ | 0.000 – . | >0.999^c^ |  |
| *Multivariate regression^d^* | | | | |  |
| Myocardial infarction | Yes vs No | **10.896** | **2.066 – 54.354** | **0.005** | ***** |
| MPN Therapy | Yes vs No | **3.592** | **1.119 – 11.533** | **0.032** | ***** |

a: Odds ratio

b: Confidence interval

c: not enough cases

d: Basic model includes: Myocardial infarction, MPN Therapy, MPN Therapy with HU , MPN Therapy with IFN

Note: The HAS-BLED score is designed to assess bleeding risk, not thrombotic risk; results should be interpreted with caution

Supplemental Table S8: Logistic regression for the thromboembolic/vascular events that occurred with/after diagnosis of MPN in patients with atrial fibrillation (n=134)

| **Risk factor** | | **OR** | **95% CI** | ***P*** |  |
| --- | --- | --- | --- | --- | --- |
| *Univariate regression* | |  |  |  |  |
| Age at first registration | 65-74 vs < 65 | 1.545 | 0.149 – 16.005 | 0.715 |  |
|  | > 74 vs < 65 | 4.587 | 0.569 – 36.966 | 0.152 |  |
| Gender female vs male | | 1.806 | 0.704 – 4.628 | 0.219 |  |
| History of Thromboembolic event Yes vs No | | 0.775 | 0.208 – 2.885 | 0.704 |  |
| History of vascular event Yes vs No | | 2.042 | 0.652 – 6.395 | 0.220 |  |
| MPN Diagnosis | PV Yes vs No | 1.114 | 0.414 – 3.002 | 0.831 |  |
|  | ET Yes vs No | 0.891 | 0.319 – 2.490 | 0.826 |  |
|  | PMF Yes vs No | 0.921 | 0.282 – 3.001 | 0.891 |  |
|  | PostPV-MF Yes vs No | 1.080 | 0.120 – 9.741 | 0.945 |  |
|  | PostET-MF Yes vs No | 0.000^a^ | 0.000 – . | 0.999^a^ |  |
|  | MPN- Unclassifiable Yes vs No | 0.515 | 0.062 – 4.251 | 0.538 |  |
|  | Others Yes vs No | 3.860 | 0.604 – 24.652 | 0.153 |  |
| Leukocyte count | < 3.5/nl vs 3.5-10/nl | 0.000^a^ | 0.000 – . | >0.999^a^ |  |
|  | > 10/nl vs 3.5-10/nl | **0.342** | **0.117 – 0.998** | **0.050** | **#** |
| Hematocrit > 45/nl vs ≤ 45/nl | | **2.700** | **1.019 – 7.155** | **0.046** | ***** |
| Platelet count | < 150/nl vs 150-450/nl | 0.000^a^ | 0.000 – . | 0.999^a^ |  |
|  | > 450/nl vs 150-450/nl | 0.868 | 0.339 – 2.226 | 0.769 |  |
| LDH > 250U/L vs ≤ 250U/L | | 1.197 | 0.420 – 3.410 | 0.737 |  |
| Myocardial infarction Yes vs No | | 2.521 | 0.593 – 10.715 | 0.210 |  |
| Congestive heart failure Yes vs No | | 0.000^a^ | 0.000 – . | 0.998^a^ |  |
| Diabetes mellitus Yes vs No | | 0.873 | 0.268 – 2.836 | 0.821 |  |
| Arterial hypertension Yes vs No | | 0.860 | 0.301 – 2.456 | 0.778 |  |
| Splenomegaly Yes vs No | | 1.128 | 0.430 – 2.9559 | 0.806 |  |
| CHA2DS2-VASc score M > 1, F > 2 vs M ≤ 1, F ≤ 2 | | 3.299 | 0.413 – 26.323 | 0.260 |  |
| Abnormal liver function Yes vs No | | 1.325 | 0.141 – 12.481 | 0.806 |  |
| Abnormal renal function | | 0.922 | 0.189 – 4.490 | 0.919 |  |
| HASBLED > 2 vs ≤ 2 | | **2.913** | **1.091 – 7.776** | **0.033** | ***** |
| Antithrombotic therapy | Yes vs No | n. a. | 0.000 – . | 0.998^a^ |  |
|  | Antiplatelet Yes vs No | **3.207** | **1.224 – 8.404** | **0.018** | ***** |
|  | Vit.K Antagonist Yes vs No | 1.168 | 0.446 – 3.057 | 0.752 |  |
|  | DOAC Yes vs No | 1.317 | 0.512 – 3.390 | 0.568 |  |
|  | Heparin Yes vs No | 1.080 | 0.120 – 9.741 | 0.945 |  |
| Driver Mutations | JAK2 Yes vs No | 1.983 | 0.538 – 7.310 | 0.304 |  |
|  | CalR Yes vs No | 2.312 | 0.186 – 28.717 | 0.514 |  |
|  | MPL Yes vs No | 2.667 | 0.231 – 30.800 | 0.432 |  |
| MPN Therapy | Yes vs No | 1.005 | 0.337 – 2.998 | 0.993 |  |
|  | Anagrelide Yes vs No | **3.031** | **1.061 – 8.664** | **0.038** | ***** |
|  | HU Yes vs No | 1.139 | 0.425 – 3.050 | 0.796 |  |
|  | RUX Yes vs No | 1.518 | 0.557 – 4.137 | 0.415 |  |
|  | IFN Yes vs No | 1.717 | 0.430 – 6.851 | 0.444 |  |
|  | Imide Yes vs No | 0.000^a^ | 0.000 – . | 0.999^a^ |  |
| *Multivariate regression^d^* | | | | |  |
| HASBLED > 2 vs ≤ 2 | | **3.346** | **1.207 – 9.272** | **0.020** | ***** |
| Antiplatelet Therapy Yes vs No | | **3.650** | **1.346 – 9.900** | **0.011** | ***** |

d Basic model includes: hematocrit, HASBLED, antiplatelet therapy and MPN therapy with Anagrelide

Supplemental Table S9: Logistic regression for thromboembolic/vascular event before diagnosis in MPN patients without atrial fibrillation (n=134)

| **Risk factor** | | **OR** | **95% CI** | ***P*** |  |
| --- | --- | --- | --- | --- | --- |
| *Univariate regression* | |  |  |  |  |
| Age at first registration | 65-74 vs < 65 | 4.690 | 0.539 – 40.801 | 0.161 |  |
|  | > 74 vs < 65 | **6.561** | **0.823 – 52.307** | **0.076** | **#** |
| Gender female vs male | | 0.672 | 0.300 – 1.504 | 0.333 |  |
| MPN Diagnosis | PV Yes vs No | 1.474 | 0.636 – 3.413 | 0.365 |  |
|  | ET Yes vs No | 0.497 | 0.195 – 1.265 | 0.142 |  |
|  | PMF Yes vs No | 1.594 | 0.658 – 3.864 | 0.302 |  |
|  | PostPV-MF Yes vs No | 0.000^a^ | 0.000 – . | >0.999^a^ |  |
|  | PostET-MF Yes vs No | 1.078 | 0.108 – 10.749 | 0.949 |  |
|  | MPN-Unclassifiable Yes vs No | 0.633 | 0.071 – 5.636 | 0.682 |  |
|  | Others Yes vs No | n.a. | n.a. | n.a. |  |
| Leukocyte count | < 3.5/nl vs 3.5-10/nl | 1.238 | 0.119 – 12.838 | 0.858 |  |
|  | > 10/nl vs 3.5-10/nl | 1.321 | 0.581 – 3.001 | 0.507 |  |
| Hematocrit > 45/nl vs ≤ 45/nl | | **2.333** | **1.005 – 5.419** | **0.049** | ***** |
| Platelet count | < 150/nl vs 150-450/nl | 1.222 | 0.317 – 4.717 | 0.771 |  |
|  | > 450/nl vs 150-450/nl | 1.222 | 0.492 – 3.036 | 0.666 |  |
| LDH ≤ 250U/L vs > 250U/L | | 0.833 | 0.320 – 2.170 | 0.709 |  |
| Myocardial infarction Yes vs No | | **20.132** | **4.021 – 100.803** | **<0.001** | * |
| Congestive heart failure Yes vs No | | 0.890 | 0.174 – 4.554 | 0.889 |  |
| Diabetes mellitus Yes vs No | | 1.759 | 0.492 – 6.290 | 0.385 |  |
| Arterial hypertension Yes vs No | | 1.412 | 0.570 – 3.496 | 0.456 |  |
| Splenomegaly Yes vs No | | 1.444 | 0.602 – 3.461 | 0.410 |  |
| CHA2DS2-VASc score  M > 1, F > 2 vs M ≤ 1, F ≤ 2 | | 705348207.742748 | 0.000 – 0. | 0.998 |  |
| Abnormal liver function | | 3.393 | 0.457 – 25.190 | 0.232 |  |
| Abnormal renal function | | 1.519 | 0.132 – 17.414 | 0.737 |  |
| HAS-BLED > 2 vs ≤ 2 | | **4.831** | **1.975 – 11.818** | **0.001** | ***** |
| Antithrombotic Therapy | Yes vs No | **2.915** | **1.032 – 8.234** | **0.043** | ***** |
|  | Antiplatelet Yes vs No | 2.052 | 0.862 – 4.883 | 0.104 |  |
|  | Vit.K Antagonist Yes vs No | 0.819 | 0.165 – 4.074 | 0.807 |  |
|  | DOAC Yes vs No | 1.759 | 0.492 – 6.290 | 0.385 |  |
|  | Heparin Yes vs No | 0.000^a^ | 0.000 – . | >0.999^a^ |  |
| Driver Mutations | Yes vs No | 0.636 | 0.056 – 7.285 | 0.211 |  |
|  | JAK2 Yes vs No | 1.421 | 0.436 – 4.626 | 0.560 |  |
|  | CalR Yes vs No | 0.000^a^ | 0.000 – . | >0.999^a^ |  |
|  | MPL Yes vs No | 0.725 | 0.072 – 7.276 | 0.785 |  |
| MPN Therapy | Yes vs No | 0.931 | 0.355 – 2.444 | 0.885 |  |
|  | Anagrelide Yes vs No | **0.138** | **0.018 – 1.076** | **0.059** | **#** |
|  | HU Yes vs No | 1.162 | 0.516 – 2.617 | 0.717 |  |
|  | RUX Yes vs No | 0.941 | 0.407 – 2.174 | 0.886 |  |
|  | IFN Yes vs No | 1.759 | 0.492 – 6.290 | 0.385 |  |
|  | Imide Yes vs No | 0.000^a^ | 0.000 – . | >0.999^a^ |  |
| *Multivariate regression^d^* | |  |  |  |  |
| Myocardial infarction Yes vs No | | **21.985** | **4.106 – 117.713** | **<0.001** | ***** |
| HASBLED > 2 vs ≤ 2 | | **4.921** | **1.742 – 13.899** | **0.003** | ***** |

d: Basic model includes: hematocrit, myocardial infarction, HAS-BLED, antithrombotic therapy,

Supplemental Table S10: Logistic regression for thromboembolic/vascular events at or after diagnosis of MPN patients without atrial fibrillation (n=134)

| **Risk factor** | | **OR** | **95% CI** | ***P*** |  |
| --- | --- | --- | --- | --- | --- |
| *Univariate regression* | |  |  |  |  |
| Age at first registration | 65-74 vs < 65 | 3.967 | 0.449 – 35.025 | 0.215 |  |
|  | > 74 vs < 65 | 2.186 | 0.259 – 18.445 | 0.472 |  |
| Gender female vs male | | 1.518 | 0.526 – 4.379 | 0.440 |  |
| History of Thromboembolic event Yes vs No | | 0.368 | 0.046 – 2.957 | 0.347 |  |
| History of vascular event Yes vs No | | 0.425 | 0.052 – 3.442 | 0.423 |  |
| MPN Diagnosis | PV Yes vs No | 1.182 | 0.405 – 3.446 | 0.760 |  |
|  | ET Yes vs No | 1.457 | 0.514 – 4.131 | 0.479 |  |
|  | PMF Yes vs No | 0.600 | 0.161 – 2.234 | 0.446 |  |
|  | PostPV-MF Yes vs No | 0.000 | 0.000 - . | 0.999 |  |
|  | PostET-MF Yes vs No | 0.000 | 0.000 - . | 0.999 |  |
|  | MPN-Unclassifiable Yes vs No | 1.362 | 0.149 – 12.423 | 0.784 |  |
|  | Others Yes vs No | n.a. | n.a. | n.a. |  |
| Leukocyte count | < 3.5/nl vs 3.5-10/nl | 0.000 | 0.000 - . | 0.999 |  |
|  | > 10/nl vs 3.5-10/nl | 1.653 | 0.586 – 4.657 | 0.342 |  |
| Hematocrit > 45/nl vs ≤ 45/nl | | 0.624 | 0.190 – 2.046 | 0.436 |  |
| Platelet count | < 150/nl vs 150-450/nl | 2.857 | 0.357 – 22.245 | 0.316 |  |
|  | > 450/nl vs 150-450/nl | **4.000** | **0.849 – 18.836** | **0.080** | **#** |
| LDH ≤ 250U/L vs > 250U/L | | 4.278 | 0.527 – 34.694 | 0.174 |  |
| Myocardial infarction Yes vs No | | 1.429 | 0.279 – 7.309 | 0.668 |  |
| Congestive heart failure Yes vs No | | 0.767 | 0.089 – 6.577 | 0.809 |  |
| Diabetes mellitus Yes vs No | | 2.571 | 0.621 – 10.642 | 0.192 |  |
| Arterial hypertension Yes vs No | | 0.452 | 0.155 – 1.316 | 0.145 |  |
| Splenomegaly Yes vs No | | 1.458 | 0.465 – 4.572 | 0.518 |  |
| CHA2DS2-VASc score  M > 1, F > 2 vs M ≤ 1, F ≤ 2 | | 3.299 | 0.413 – 26.323 | 0.260 |  |
| Abnormal liver function | | 1.325 | 0.141 – 12.481 | 0.806 |  |
| Abnormal renal function | | 0.922 | 0.189 – 4.490 | 0.919 |  |
| HASBLED > 2 vs ≤ 2 | | **2.913** | **1.091 – 7.776** | **0.033** | * |
| Antithrombotic therapy | Yes vs No | 360903911 | 0.000 - . | 0.998 |  |
|  | Antiplatelet Yes vs No | **3.207** | **1.224 – 8.404** | **0.018** | ***** |
|  | Vit.K Antagonist Yes vs No | 1.168 | 0.446 – 3.057 | 0.752 |  |
|  | DOAC Yes vs No | 1.317 | 0.512 – 3.390 | 0.568 |  |
|  | Heparin Yes vs No | 1.080 | 0.120 – 9.741 | 0.945 |  |
| Driver Mutations | JAK2 Yes vs No | 1.983 | 0.538 – 7.310 | 0.304 |  |
|  | CalR Yes vs No | 2.312 | 0.186 – 28.717 | 0.514 |  |
|  | MPL Yes vs No | 2.667 | 0.231 – 30.800 | 0.432 |  |
| MPN Therapy | Yes vs No | 1.005 | 0.337 – 2.998 | 0.993 |  |
|  | Anagrelide Yes vs No | **3.031** | **1.061 – 8.664** | **0.038** | ***** |
|  | HU Yes vs No | 1.139 | 0.425 – 3.050 | 0.796 |  |
|  | RUX Yes vs No | 1.518 | 0.557 – 4.137 | 0.415 |  |
|  | IFN Yes vs No | 1.717 | 0.430 – 6.851 | 0.444 |  |
|  | Imide Yes vs No | 0.000 | 0.000 - . | 0.999 |  |
| *Multivariate regression^d^* | |  |  |  |  |
| MPN Therapy | Anagrelide Yes vs No | **3.709** | **1.195 – 11.514** | **0.023** | ***** |

d: Basic model includes: HASBLED, Antiplatelet therapy, MPN therapy with Anagrelide,

Supplemental Table S11: Logistic regression for thromboembolic/vascular events that occurred with/after diagnosis of MPN in all patients (n=2,780)

| **Risk factor** | | **OR** | **95% CI** | ***P*** |  |
| --- | --- | --- | --- | --- | --- |
| *Univariate regression* | | | | |  |
| Age at first registration | 65-74 vs <65 | 1.042 | 0.793 – 1.369 | 0.768 |  |
|  | >74vs <65 | 1.157 | 0.888 – 1.507 | 0.280 |  |
| Gender female vs male | | 0.949 | 0.763 – 1.179 | 0.653 |  |
| History of thromboembolic event Yes vs No | | 1.023 | 0.742 – 1.410 | 0.889 |  |
| History of vascular event Yes vs No | | **1.600** | **1.137 – 2.252** | **0.007** | ***** |
| MPN Diagnosis | PV Yes vs No | **1.604** | **1.280 – 2.010** | **<0.001** | ***** |
|  | ET Yes vs No | 0.960 | 0.763 – 1.209 | 0.731 |  |
|  | PMF Yes vs No | **0.713** | **0.536 – 0.947** | **0.019** | ***** |
|  | PostPV-MF Yes vs No | 0.767 | 0.365 – 1.612 | 0.484 |  |
|  | PostET-MF Yes vs No | **0.399** | **0.161 – 0.994** | **0.048** |  |
|  | MPN-Unclassifiable Yes vs No | 0.707 | 0.410 – 1.220 | 0.213 |  |
|  | Others Yes vs No | 0.928 | 0.438 – 1.966 | 0.845 |  |
| Leukocyte count | < 3.5/nl vs 3.5-10/nl | 0.836 | 0.412 – 1.700 | 0.621 |  |
|  | > 10/nl vs 3.5-10/nl | 1.177 | 0.940 – 1.474 | 0.156 |  |
| Hematocrit > 45/nl vs ≤ 45/nl | | 1.143 | 0.900 – 1.452 | 0.272 |  |
| Platelet count | < 150/nl vs 150-450/nl | 0.688 | 0.450 – 1.051 | 0.083 | # |
|  | > 450/nl vs 150-450/nl | **0.714** | **0.566 – 0.902** | **0.005** | ***** |
| LDH > 250U/L vs ≤ 250U/L | | 0.924 | 0.727 – 1.174 | 0.518 |  |
| Myocardial infarction Yes vs No | | **3.649** | **2.535 – 5.253** | **<0.001** | ***** |
| Congestive heart failure Yes vs No | | 1.294 | 0.858 – 1.952 | 0.219 |  |
| Diabetes mellitus Yes vs No | | 1.122 | 0.766 – 1.644 | 0.554 |  |
| Arterial hypertension Yes vs No | | 1.214 | 0.965 – 1.527 | 0.098 |  |
| Splenomegaly Yes vs No | | 1.259 | 0.099 – 1.588 | 0.051 | # |
| CHA2DS2-VASc score M > 1, F > 2 vs M ≤ 1, F ≤ 2 | | 1.165 | 0.937 – 1.449 | 0.169 |  |
| Abnormal liver function Yes vs No | | **2.865** | **1.782 – 4.605** | **<0.001** | ***** |
| Abnormal renal function Yes vs No | | 1.407 | 0.864 – 2.293 | 0.170 |  |
| HASBLED > 2 vs ≤ 2 | | **2.183** | **1.722 – 2.768** | **<0.001** | ***** |
| Antithrombotic therapy | Yes vs No | **3.928** | **2.861 – 5.392** | **<0.001** | ***** |
|  | Antiplatelet Yes vs No | **1.479** | **1.178 – 1.856** | **0.001** | ***** |
|  | Vit.K Antagonist Yes vs No | **4.750** | **3.481 – 6.481** | **<0.001** | ***** |
|  | DOAC Yes vs No | **3.064** | **2.181 – 4.305** | **<0.001** | ***** |
|  | Heparin Yes vs No | **4.025** | **2.508 – 6.458** | **<0.001** | ***** |
| Driver Mutations | JAK2 Yes vs No | **1.478** | **1.118 – 1.954** | **0.006** | ***** |
|  | CalR Yes vs No | 0.662 | 0.413 – 1.060 | 0.086 | # |
|  | MPL Yes vs No | 1.328 | 0.637 – 2.767 | 0.449 |  |
| MPN Therapy | Yes vs No | **2.842** | **2.073 – 3.895** | **<0.001** | ***** |
|  | Anagrelide Yes vs No | **1.722** | **1.323 – 2.242** | **<0.001** | ***** |
|  | HU Yes vs No | **1.920** | **1.529 – 2.410** | **<0.001** | ***** |
|  | RUX Yes vs No | **1.371** | **1.086 – 1.730** | **0.008** | ***** |
|  | IFN Yes vs No | **1.872** | **1.391 – 2.519** | **<0.001** | ***** |
|  | Imide No vs Yes | 0.000^a^ | 0.000 – . | 0.998^a^ |  |
| *Multivariate regression^d^* | | | | |  |
| Platelet count | > 450/nl vs 150-450/nl | **0.677** | **0.519 – 0.883** | **0.004** | ***** |
| Myocardial infarction Yes vs No | | **3.307** | **2.231 – 4.901** | **<0.001** | ***** |
| Antithrombotic therapy | Yes vs No | **2.519** | **1.722 – 3.687** | **<0.001** | ***** |
|  | Vit.K Antagonist Yes vs No | **3.584** | **2.507 – 5.122** | **<0.001** | ***** |
|  | DOAC Yes vs No | **2.441** | **1.657 – 3.595** | **<0.001** | ***** |
|  | Heparin Yes vs No | **2.72/** | **1.606 – 4.637** | **<0.001** | ***** |
| MPN Therapy | Yes vs No | **1.819** | **1.276 – 2.594** | **0.001** | ***** |
|  | Anagrelide Yes vs No | **1.726** | **1.258 – 2.369** | **0.001** | ***** |

d: Basic model includes: MPN Diagnosis (PV, PMF, PostET-MF), myocardial infarction, HASBLED, history of vascular event, anticoagulation therapy, Antiplatelet therapy, VKA, DOAC, Heparin, JAK2 mutation, MPN therapy, MPN therapy with Anagrelide, HU, RUX and IFN, Abnormal liver function, , Platelet count

Supplemental Table S12: Logistic regression for bleeding events that occurred with/after diagnosis of MPN in patients with atrial fibrillation (n=134)

| **Risk factor** | | **OR** | **95% CI** | ***P*** |  |
| --- | --- | --- | --- | --- | --- |
| *Univariate regression* | |  |  |  |  |
| Age at first registration | 65-74 vs < 65 | 1.454 | 0.149 – 16.005 | 0.715 |  |
|  | > 74vs < 65 | 1630 | 0.188 – 14.146 | 0.658 |  |
| Gender female vs male | | 1.030 | 0.298 – 3.556 | 0.962 |  |
| MPN Diagnosis | PV Yes vs No | 1.278 | 0.353 – 4.628 | 0.708 |  |
|  | ET Yes vs No | 0.208 | 0.026 – 1.678 | 0.140 |  |
|  | PMF Yes vs No | 0.871 | 0.177 – 4.288 | 0.865 |  |
|  | PostPV-MF Yes vs No | **6.611** | **1.063 – 41.115** | **0.043** | ***** |
|  | PostET-MF Yes vs No | 0.000^c^ | 0.000 – . | 0.999^c^ |  |
|  | MPN-Unclassifiable Yes vs No | 1.130 | 0.131 – 9.749 | 0.911 |  |
|  | Others Yes vs No | 2.975 | 0.303 – 29.212 | 0.350 |  |
| Leukocyte count | < 3.5/nl vs 3.5-10/nl | 0.000^c^ | 0.000 – . | 0.999^c^ |  |
|  | > 10/nl vs 3.5-10/nl | 1.592 | 0.461 – 5.503 | 0.462 |  |
| Hematocrit >45/nl vs ≤45/nl | | 1.957 | 0.565 – 6.776 | 0.290 |  |
| Platelet count | < 150/nl vs 150-450/nl | 1.815 | 0.315 – 10.455 | 0.505 |  |
|  | >450/nl vs 150-450/nl | 0.377 | 0.090 – 1.582 | 0.183 |  |
| LDH > 250U/L vs ≤ 250U/L | | 2.599 | 0.535 – 12.612 | 0.236 |  |
| Myocardial infarction Yes vs No | | 1.189 | 0.136 – 10.363 | 0.876 |  |
| Congestive heart failure Yes vs No | | 0.333 | 0.041 – 2.722 | 0.305 |  |
| Diabetes mellitus Yes vs No | | 0.829 | 0.169 – 4.074 | 0.817 |  |
| Arterial hypertension Yes vs No | | 0.441 | 0.111 – 1.752 | 0.245 |  |
| Splenomegaly Yes vs No | | 0.989 | 0.214 – 3.771 | 0.884 |  |
| Abnormal liver function Yes vs No | | 2.900 | 0.295 – 2.481 | 0.361 |  |
| Abnormal renal function Yes vs No | | 3.511 | 0.811 – 15.201 | 0.093 |  |
| CHA2DS2-VASc score M > 1, F > 2 vs M ≤ 1, F ≤ 2 | | 0.343 | 0.081 – 1.444 | 0.144 |  |
| HASBLED > 2 vs ≤ 2 | | 1.030 | 0.298 – 3.556 | 0.962 |  |
| Antithrombotic therapy | Yes vs No | 1.714 | 0.207 – 14.219 | 0.618 |  |
|  | Antiplatelet Yes vs No | 2.080 | 0.601 – 7.204 | 0.248 |  |
|  | Vit.K Antagonist Yes vs No | **5.744** | **1.445 – 22.83** | **0.013** | ***** |
|  | DOAC Yes vs No | 0.347 | 0.072 – 1.676 | 0.188 |  |
|  | Heparin Yes vs No | 2.360 | 0.251 – 22.212 | 0.453 |  |
| Driver Mutations | JAK2 Yes vs No | 3.690 | 0.454 – 30.031 | 0.222 |  |
|  | CalR Yes vs No | 0.000^c^ | 0.000 – . | 0.999^c^ |  |
|  | MPL Yes vs No | 0.000^c^ | 0.000 - . | 0.999^c^ |  |
| MPN Therapy | Yes vs No | 0.823 | 0.205 – 3.305 | 0.783 |  |
|  | Anagrelide Yes vs No | 1.079 | 0.217 – 5.360 | 0.926 |  |
|  | HU Yes vs No | 0.432 | 0.125 – 1.499 | 0.186 |  |
|  | RUX Yes vs No | 1.696 | 0.465 – 6.186 | 0.424 |  |
|  | IFN Yes vs No | 0.925 | 0.109 – 7.863 | 0.943 |  |
|  | Imide No vs Yes | 6.050 | 0.504 – 72.649 | 0.156 |  |
| *Multivariate regression* | |  |  |  |  |
| Post PVMF | Yes vs No | **8.816** | **1.150 – 67.576** | **0.036** | ***** |
| Anticoagulationtherapy | Vit. K Antagonist Yes vs No | **6.455** | **1.523 – 27.360** | **0.011** | ***** |

d: Basic model includes: PostPV-MF, VKA

Supplemental Table S13: Logistic regression for bleeding events that occurred with/after diagnosis of MPN in patients without atrial fibrillation, age matched (n=134)

| **Risk factor** | | **OR** | **95% CI** | ***P*** |  |
| --- | --- | --- | --- | --- | --- |
| *Univariate regression* | |  |  |  |  |
| Age at first registration | 65-74 vs < 65 | 0.222 | 0.019 – 2.632 | 0.233 |  |
|  | > 74 vs < 65 | 0.316 | 0.049 – 2.046 | 0.227 |  |
| Gender female vs male | | 0.377 | 0.067 – 2.131 | 0.270 |  |
| MPN Subtypes | PV Yes vs No | 0.410 | 0.046 – 3.622 | 0.422 |  |
|  | ET Yes vs No | 0.988 | 0.174 – 5.615 | 0.989 |  |
|  | PMF Yes vs No | 3.167 | 0.607–16.524 | 0.171 |  |
|  | PostPV-MF Yes vs No | 0.000 | 0.000 - . | 0.999^c^ |  |
|  | PostET-MF Yes vs No | 0.000 | 0.000 - . | 0.999^c^ |  |
|  | MPN-Unclassifiable Yes vs No | 0.000 | 0.000 - . | 0.999^c^ |  |
|  | Others Yes vs No | 0.539 | 0.074 – 3.940 | 0.542 |  |
| Leukocyte count | < 3.5/nl vs 3.5-10/nl | 0.000 | 0.000 - . | 0.999^c^ |  |
|  | > 10/nl vs 3.5-10/nl | 5.804 | 0.658 – 51.164 | 0.113 |  |
| Hematocrit > 45 /nl vs ≤ 45 /nl | | 0.415 | 0.047 – 3.617 | 0.429 |  |
| Platelet count | < 150/nl vs 150-450/nl | 1.333 | 0.112 – 15.806 | 0.820 |  |
|  | > 450/nl vs 150-450/nl | 0.870 | 0.139 – 5.427 | 0.881 |  |
| LDH > 250 U/L vs ≤ 250 U/L | | 0.506 | 0.080 – 3.193 | 0.469 |  |
| Myocardial infarction | | 1.980 | 0.210 – 18.662 | 0.551 |  |
| Congestive heart failure Yes vs No | | 0.000 | 0.000 - . | 0.999^c^ |  |
| Diabetes mellitus Yes vs No | | 0.000 | 0.000 - . | 0.999^c^ |  |
| Arterial hypertension Yes vs No | | 0.977 | 0.157 – 6.092 | 0.980 |  |
| Splenomegaly Yes vs No | | 3.672 | 0.415 – 32.476 | 0.242 |  |
| Abnormal liver function Yes vs No | | 0.000 | 0.000 - . | 0.999^c^ |  |
| Abnormal renal function Yes vs No | | 0.000 | 0.000 - . | 0.999^c^ |  |
| CHA2DS2-VASc score M > 1, F > 2 vs M ≤ 1, F ≤ 2 | | 0.293 | 0.056 – 1.530 | 0.145 |  |
| HASBLED > 2 vs ≤ 2 | | 0.714 | 0.080 – 6.367 | 0.763 |  |
| Antithrombotic therapy | Yes vs No | 2.356 | 0.267 – 20.822 | 0.441 |  |
|  | Antiplatelet Yes vs No | 1.459 | 0.258 – 8.259 | 0.669 |  |
|  | Vit.K Antagonist Yes vs No | **7.500** | **1.189 – 47.323** | **0.032** | ***** |
|  | DOAC Yes vs No | 2.127 | 0.228 – 19.867 | 0.508 |  |
|  | Heparin Yes vs No | 0.000 | 0.000 - . | 0.999^c^ |  |
| Driver Mutations | JAK2 Yes vs No | 0.969 | 0.107 – 8.771 | 0.978 |  |
|  | CalR Yes vs No | 179497205 | 0.000 - . | 0.998 |  |
|  | MPL Yes vs No | 0.000 | 0.000 - . | 0.999^c^ |  |
| MPN Therapy | Yes vs No | 97907567.9 | 0.000 - . | 0.998 |  |
|  | Anagrelide Yes vs No | 1.080 | 0.120 – 9.741 | 0.945 |  |
|  | HU Yes vs No | 0.803 | 0.156 – 4.130 | 0.793 |  |
|  | RUX Yes vs No | 3.689 | 0.650 – 20.927 | 0.140 |  |
|  | IFN Yes vs No | 2.127 | 0.228 – 19.867 | 0.508 |  |
|  | Imide Yes vs No | 0.000 | 0.000 - . | 0.999^c^ |  |
| *Multivariate regression* | |  |  |  |  |

Supplemental Table S14: Logistic regression for bleeding events that occurred with/after diagnosis of MPN in all patients (n=2,780)

| **Risk factor** | | **OR** | **95% CI** | ***P*** |  |
| --- | --- | --- | --- | --- | --- |
| *Univariate regression* | |  |  |  |  |
| Age at first registration | 65-74 vs < 60 | 0.832 | 0.482 – 1.438 | 0.511 |  |
|  | > 74 vs < 60 | 1.193 | 0.736 – 1.934 | 0.474 |  |
| Gender female vs male | | **0.514** | **0.334 – 0.790** | **0.002** | ***** |
| MPN Diagnosis | PV Yes vs No | 1.377 | 0899 – 2.109 | 0.141 |  |
|  | ET Yes vs No | 0.341 | 0.513 – 1.260 | 0.341 |  |
|  | PMF Yes vs No | 0.957 | 0.579 – 1.582 | 0.864 |  |
|  | PostPV-MF Yes vs No | 1.652 | 0.590 – 4.626 | 0.339 |  |
|  | PostET-MF Yes vs No | 0.339 | 0.047 – 2.462 | 0.285 |  |
|  | MPN-Unclassifiable Yes vs No | 0.780 | 0.282 – 2.154 | 0.632 |  |
|  | Others Yes vs No | 0.933 | 0.225 – 3.874 | 0.924 |  |
| Leukocyte count | < 3.5/nl vs 3.5-10/nl | 1.988 | 0.692 – 5.711 | 0.202 |  |
|  | > 10/nl vs 3.5-10/nl | **1.891** | **1.233 – 2.900** | **0.009** | ***** |
| Hematocrit > 45/nl vs ≤ 45/nl | | 1.029 | 0.655 – 1.618 | 0.900 |  |
| Platelet count | < 150/nl vs 150-450/nl | 1.200 | 0.602 – 2.393 | 0.605 |  |
|  | > 450/nl vs 150-450/nl | 0.844 | 0.541 – 1.315 | 0.453 |  |
| LDH > 250U/L vs ≤ 250U/L | | **2.250** | **1.355 – 3.737** | **0.002** | ***** |
| Myocardial infarction | | 1.852 | 0.909 – 3.772 | 0.0895 | # |
| Congestive heart failure Yes vs No | | 1.114 | 0.506 – 2.452 | 0.788 |  |
| Diabetes mellitus Yes vs No | | 0.483 | 0.176 – 1.328 | 0.158 |  |
| Arterial hypertension Yes vs No | | 0.928 | 0.597 – 1.443 | 0.741 |  |
| Splenomegaly Yes vs No | | 1.378 | 0.883 – 2.149 | 0.158 |  |
| Abnormal liver function Yes vs No | | **3.508** | **1.698 – 7.247** | **0.001** | ***** |
| Abnormal renal function Yes vs No | | 2.101 | 0.989 – 4.464 | 0.053 | # |
| CHA2DS2-VASc score M > 1, F > 2 vs M ≤ 1, F ≤ 2 | | 0.853 | 0.561 – 1.296 | 0.456 |  |
| HASBLED > 2 vs ≤ 2 | | **1.645** | **1.047 – 2.585** | **0.031** | ***** |
| Antithrombotic therapy | Yes vs No | 1.530 | 0.949 – 2.465 | 0.081 | # |
|  | Antiplt. Yes vs No | 1.154 | 0.758 – 1.758 | 0.505 |  |
|  | Vit.K Antagonist Yes vs No | **3.052** | **1.767 – 5.271** | **<0.001** | ***** |
|  | DOAC Yes vs No | 1.565 | 0.774 – 3.166 | 0.213 |  |
|  | Heparin Yes vs No | **4.011** | **1.938 – 8.302** | **<0.001** | ***** |
| Driver Mutations | JAK2 Yes vs No | 1.327 | 0.793 – 2.223 | 0.282 |  |
|  | CalR Yes vs No | 1.102 | 0.352 – 3.444 | 0.868 |  |
|  | MPL Yes vs No | 1.262 | 0.163 – 9.766 | 0.823 |  |
| MPN Therapy | Yes vs No | **4.148** | **2.000 – 8.602** | **<0.001** | ***** |
|  | Anagrelide Yes vs No | **1.811** | **1.126 – 2.913** | **0.014** | ***** |
|  | HU Yes vs No | **1.740** | **1.132 – 2.676** | **0.012** | ***** |
|  | RUX Yes vs No | **2.494** | **1.649 – 3.772** | **<0.001** | ***** |
|  | IFN Yes vs No | **1.925** | **1.135 – 3.263** | **0.015** | ***** |
|  | Imide Yes vs No | 1.922 | 0.453 – 8.166 | 0.376 |  |
| *Multivariate regression* | |  |  |  |  |
| Gender female vs male | | **0.514** | **0.334 – 0.790** | **0.002** | ***** |
| LDH > 250U/L vs ≤ 250U/L | | **2.250** | **1.355 – 3.737** | **0.002** | ***** |
| Abnormal liver function Yes vs No | | **3.508** | **1.698 – 7.247** | **0.001** | ***** |
| Antithrombotic therapy | Vit. K Antagonist Yes vs No | **5.171** | **1.439 – 18.578** | **0.012** | ***** |
|  | Heparin Yes vs No | **4.011** | **1.938 – 8.302** | **<0.001** | ***** |
| MPN Therapy | Anagrelide Yes vs No | **1.811** | **1.126 – 2.913** | **0.014** | ***** |
|  | HU Yes vs No | **1.740** | **1.132 – 2.676** | **0.012** | ***** |
|  | RUX Yes vs No | **2.494** | **1.649 – 3.772** | **<0.001** | ***** |

d: Basic model includes: gender, leukocyte count, LDH, Abnormal liver function, HASBLED, Vit. K Antagonist, Heparin, MPN therapy, MPN therapy withAnagrelide, HU, RUX and IFN

### Discussion regarding logistic regression analyses

De Freitas et al. compared 62 PV pts with coexisting AF (median age 73) with 124 AF pts without MPN (median age 77). In multivariate analysis, PV pts with AF did not show any increased risk of thrombosis or bleeding compared to control group after correction by age, sex, history of thrombosis and smoking ^11^.

Meanwhile, AF was associated with higher risk of TE in the analysis from Mahé et al, where 713 PV/ET pts were analyzed, among whom 96 pts had coexisting AF. In this multivariate analysis, AF was associated with higher risk of thrombosis independently from age, CVRF and use of cytoreductive therapies. Case-control study from Mahé et al matching age, sex, MPN subtypes and driver mutations showed higher frequency of TE (especially for arterial TE) before and after MPN diagnosis.

Several studies have identified female sex as an additional risk factor for TE in pts with AF ^22, 29^. On the other hand, De Freitas et al and Carobbio et al showed a higher probability of bleeding in male MPN pts ^11, 30^. In our GSG-MPN bioregistry cohort, sex was not identified as a risk factor for TE or bleeding events in MPN. The insignificant effect of sex could be due to small sample size, due to the sex adjustment and management according to CHA2S2-VASc scoring system in AF pts, or due to the characteristics of MPN.

The insignificant predictive value of CHA2DS2-VASc score for predicting risk of TE could be due to prophylactic ATTs according to their risk stratification. This could be seen with the higher rate of treatment with ATTs in pts with higher CHA2DS2-VASc score (Supplemental Table S2). Likewise, the CHA2DS2-VASc score did not show prognostic value for TE occurring after MPN diagnosis, possibly due to MPN treatments.

Significantly elevated OR in high HAS-BLED score for TE could be influenced by many factors. Recent data suggest that abnormal liver function, which is one of the HAS-BLED criteria, could be related to increase in TE as much as to increase in bleeding ^31-33^. This may be related to the fact that liver disease can lead to defects in both procoagulant and anticoagulant pathways ^34^. With the exception of factor VIII and vWF, both procoagulant and anticoagulant factors are predominantly synthesized in the liver and are frequently decreased in liver disease.

Arterial hypertension, age > 65 years, and prior history of stroke are also criteria included in the HAS-BLED scoring system, thus they have been identified as risk factors both for TE and BE. It should be noted that use of ATT is one of the criteria of HAS-BLED score. Significantly increased OR in APA could have influenced the increased OR in high HAS-BLED score in multivariate analysis for TE in AF pts. Due to missing data regarding the timepoint between the initiation of ATT and the occurrence of thromboembolism, a causative link between these two factors cannot be confirmed. It should be mentioned that data for alcohol- or drug abuse were not sufficiently documented in this bioregistry, and, thus, the scoring for HAS-BLED could be underestimated.

The difference between PMF, postPV-MF, and postET-MF is also quite interesting. Interestingly, in pts with AF, postPV-MF was identified as a strong independent risk factor for bleeding. This could be due to the fact that postPV-MF is a highly thrombogenic disease ^23^, which could have influenced the indication of ATTs. This significant increase in risk of BE was identified only in pts with AF. This can be explained by more frequent presence of an indication for ATTs in this pt group.

Leukocytosis emerged as a risk factor for both TE and BE in numerous studies ^35-41^, and it is proposed to be included in the thromboembolic risk assessment ^35, 42^ . In our cohort, leukocytosis trended to act as a risk factor for TE in AF pts, but therapy-associated TE due to disease progression should also be considered.

Treatment with VKA was shown to be an independent risk factor for bleeding. This may be due to frequent continuous administration of VKA parallel to cytoreductive therapies. The majority of the pts with AF in our study were continued with ATTs (with VKA being the most frequently used type of anticoagulation) during cytoreductive therapies.

# Supplemental Figures

Supplemental Figure S1: Prevalence of atrial fibrillation according to age in patients with MPN. Percentage of AF in each age group: 40-49 years (0.3%), 50-59 years (1.2%), 60-69 years (3.6%), 70-79 years (8.7%), 80-89 years (18.2%).

|  |  |
| --- | --- |

Supplemental Figure S2: Age distribution.

| A)  B) |
| --- |
| Supplemental Figure S3: (A) Number of Thromboembolic events over time with relation to the date of diagnosis (time point “zero”). The frequency of TE was highest within 1 years prior to or following MPN diagnosis with similar distribution before or after the date of diagnosis. (B) Incidence of major bleeding events over time in respect to the time point of first MPN diagnosis. Although bleeding events were also shown to occur close to the date of diagnosis, unlike in cases of TE, most of the bleeding events occurred after the diagnosis of MPN. |

| A | B | C |
| --- | --- | --- |
|  |  |  |

Supplemental Figure S4: Survival analyses in the entire cohort (n=2,780 pts). (A) OS of pts with MPN, Cox regression analyses: for AF: HR =1.961, 95% CI = 1.34 – 2.86, P = 0.001. (B) TE-free survival, Cox regression analyses: for AF: HR =1.108, 95% CI = 0.705 – 1.740, P = 0.657. (C) Bleeding event-free survival, Cox regression analyses: for AF: HR = 2.461, 95% CI = 1.31 – 4.62, P = 0.005.

| A | B | C |
| --- | --- | --- |
|  |  |  |

Supplemental Figure S5 Survival analyses of non-AF pts stratified by antithrombotic and cytoreductive therapy, age-matched with AF pts (n=134) (A) OS of MPN pts without AF with antithrombotic or cytoreductive therapy, neither or both. Cox regression analyses: antithrombotic therapies vs both (HR=0.405, 95% CI 0.19 – 0.85, P= 0.017) Cytoreductive therapies vs (HR=1.19, 95% CI 0.49 – 2.94, P= 0.700) (B) TE-free Survival of MPN pts without AF with antithrombotic or cytoreductive therapy, neither or both. (C) Bleeding-free survival of MPN pts without AF with antithrombotic or cytoreductive therapy, neither or both.

|  | | | |
| --- | --- | --- | --- |
| A |  | B |  |

Supplemental Figure S6: OS stratified by antithrombotic and cytoreductive therapy, age-matched. (A) MPN pts with or without AF and with or without antithrombotic therapies. Cox regression analyses: atrial fibrillation (HR =1.164, 95% CI 0.685 – 1.979, P=0.574) antithrombotic therapies (HR=1.825, 95% CI 0.991 – 3.361, P= 0.053). (B) MPN pts with or without AF and with or without MPN-specific cytoreductive therapies.

| A |  | B |  |
| --- | --- | --- | --- |

Supplemental Figure S7: TE-free survival stratified by antithrombotic and cytoreductive therapy. (A) MPN pts with or without AF and with or without antithrombotic therapies. (B) MPN pts with or without AF and with or without MPN-specific cytoreductive therapies. Cox regression analyses: atrial fibrillation (HR =1.008, 95% CI 0.57 – 2.08, P=0.800), Cytoreductive therapies (HR=0.428 95% CI 0.17 – 1.06, P= 0.068).

| A |  | B |  |
| --- | --- | --- | --- |

Supplemental Figure S8: Bleeding-free survival stratified by antithrombotic and cytoreductive therapy(A) MPN pts with or without AF and with or without antithrombotic therapies. (B) MPN pts with or without AF and with or without MPN-specific cytoreductive therapies. Cox regression analyses: atrial fibrillation (HR = 1.504, 95% CI 0.56 – 4.07, P = 0.422), Cytoreductive therapies (HR = 0.454 95% CI 0.13 – 1.64, P= 0.228).


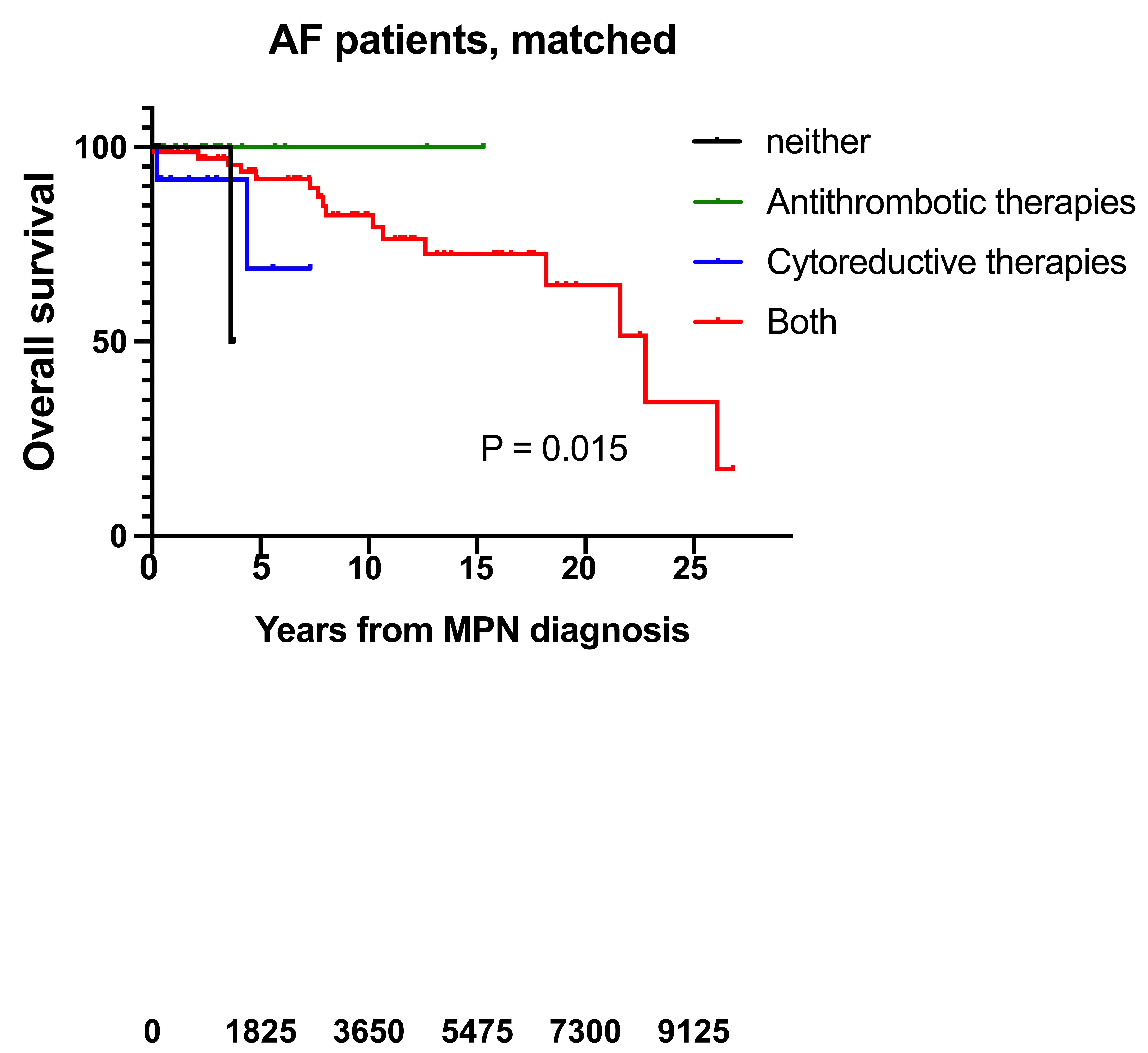


Supplemental Figure S9: OS stratified by antithrombotic and cytoreductive therapy in MPN pts with AF with antithrombotic or cytoreductive therapy, neither or both :Matched with age, congestive heart failure, diabetes mellitus arterial hypertension, abnormal renal function, history of thromboembolic and vascular events). Cox regression analyses: neither vs both (HR=9.388 95% CI 0.987– 89.257, P= 0.051) antithrombotic therapies vs both (not applicable since number of events=0) Cytoreductive therapies vs both (HR=4.182, 95% CI 0.816 – 21.436, P= 0.086)

**Supplementary References**

1 Rungjirajittranon T, Owattanapanich W, Ungprasert P, Siritanaratkul N, Ruchutrakool T. A systematic review and meta-analysis of the prevalence of thrombosis and bleeding at diagnosis of Philadelphia-negative myeloproliferative neoplasms. *BMC Cancer*. 2019; **19**: 184.

2 Barbui T, Finazzi G, Falanga A. Myeloproliferative neoplasms and thrombosis. *Blood*. 2013; **122**: 2176-84.

3 Kroll MH, Michaelis LC, Verstovsek S. Mechanisms of thrombogenesis in polycythemia vera. *Blood Rev*. 2015; **29**: 215-21.

4 Appelmann I, Kreher S, Parmentier S, Wolf H-H, Bisping G, Kirschner M*, et al.* Diagnosis, prevention, and management of bleeding episodes in Philadelphia-negative myeloproliferative neoplasms: recommendations by the Hemostasis Working Party of the German Society of Hematology and Medical Oncology (DGHO) and the Society of Thrombosis and Hemostasis Research (GTH). *Annals of Hematology*. 2016; **95**: 707-18.

5 Vener C, Artoni A, Boschetti C, Fracchiolla NS, Gianelli U, Cortelezzi A*, et al.* An acquired factor VIII inhibitor in a myeloproliferative neoplasm presenting with severe retroperitoneal hemorrhage. *Leukemia & Lymphoma*. 2012; **53**: 2296-98.

6 Camm AJ, Kirchhof P, Lip GYH, Schotten U, Savelieva I, Ernst S*, et al.* Guidelines for the management of atrial fibrillation: The Task Force for the Management of Atrial Fibrillation of the European Society of Cardiology (ESC). *European Heart Journal*. 2010; **31**: 2369-429.

7 Camm AJ, Lip GYH, De Caterina R, Savelieva I, Atar D, Hohnloser SH*, et al.* 2012 focused update of the ESC Guidelines for the management of atrial fibrillation. *European Heart Journal*. 2012; **33**: 2719-47.

8 Enga KF, Rye-Holmboe I, Hald EM, Løchen ML, Mathiesen EB, Njølstad I*, et al.* Atrial fibrillation and future risk of venous thromboembolism:the Tromsø study. *Journal of Thrombosis and Haemostasis*. 2015; **13**: 10-16.

9 Noel P, Gregoire F, Capon A, Lehert P. Atrial fibrillation as a risk factor for deep venous thrombosis and pulmonary emboli in stroke patients. *Stroke*. 1991; **22**: 760-2.

10 Mahé K, Delluc A, Chauveau A, Castellant P, Mottier D, Dalbies F*, et al.* Incidence and impact of atrial arrhythmias on thrombotic events in MPNs. *Annals of Hematology*. 2018; **97**: 101-07.

11 De Freitas AS, Alvarez-Larrán A. Risk of thrombosis and hemorrhage in patients with polycythemia vera and atrial fibrillation treated with prophylactic oral anticoagulants. *Annals of Hematology*. 2016; **95**: 1903-04.

12 Vardiman JW, Thiele J, Arber DA, Brunning RD, Borowitz MJ, Porwit A*, et al.* The 2008 revision of the World Health Organization (WHO) classification of myeloid neoplasms and acute leukemia: rationale and important changes. *Blood*. 2009; **114**: 937-51.

13 Arber DA, Orazi A, Hasserjian R, Thiele J, Borowitz MJ, Le Beau MM*, et al.* The 2016 revision to the World Health Organization classification of myeloid neoplasms and acute leukemia. *Blood*. 2016; **127**: 2391-405.

14 Lip GY, Nieuwlaat R, Pisters R, Lane DA, Crijns HJ. Refining clinical risk stratification for predicting stroke and thromboembolism in atrial fibrillation using a novel risk factor-based approach: the euro heart survey on atrial fibrillation. *Chest*. 2010; **137**: 263-72.

15 Pisters R, Lane DA, Nieuwlaat R, De Vos CB, Crijns HJGM, Lip GYH. A Novel User-Friendly Score (HAS-BLED) To Assess 1-Year Risk of Major Bleeding in Patients With Atrial Fibrillation. *Chest*. 2010; **138**: 1093-100.

16 January CT, Wann LS, Calkins H, Chen LY, Cigarroa JE, Cleveland JC*, et al.* 2019 AHA/ACC/HRS Focused Update of the 2014 AHA/ACC/HRS Guideline for the Management of Patients With Atrial Fibrillation: A Report of the American College of Cardiology/American Heart Association Task Force on Clinical Practice Guidelines and the Heart R. *Circulation*. 2019; **140**.

17 Anderson FA, Jr., Spencer FA. Risk factors for venous thromboembolism. *Circulation*. 2003; **107**: I9-16.

18 Barbui T, Carobbio A, Cervantes F, Vannucchi AM, Guglielmelli P, Antonioli E*, et al.* Thrombosis in primary myelofibrosis: incidence and risk factors. *Blood*. 2010; **115**: 778-82.

19 Rupoli S, Goteri G, Picardi P, Micucci G, Canafoglia L, Scortechini AR*, et al.* Thrombosis in essential thrombocytemia and early/prefibrotic primary myelofibrosis: the role of the WHO histological diagnosis. *Diagnostic Pathology*. 2015; **10**.

20 Lip GY. Implications of the CHA(2)DS(2)-VASc and HAS-BLED Scores for thromboprophylaxis in atrial fibrillation. *Am J Med*. 2011; **124**: 111-4.

21 Feinberg WM, Blackshear JL, Laupacis A, Kronmal R, Hart RG. Prevalence, age distribution, and gender of patients with atrial fibrillation. Analysis and implications. *Arch Intern Med*. 1995; **155**: 469-73.

22 Kannel WB, Abbott RD, Savage DD, McNamara PM. Epidemiologic features of chronic atrial fibrillation: the Framingham study. *N Engl J Med*. 1982; **306**: 1018-22.

23 Kaifie A, Kirschner M, Wolf D, Maintz C, Hanel M, Gattermann N*, et al.* Bleeding, thrombosis, and anticoagulation in myeloproliferative neoplasms (MPN): analysis from the German SAL-MPN-registry. *J Hematol Oncol*. 2016; **9**: 18.

24 Hultcrantz M, Björkholm M, Dickman PW, Landgren O, Derolf Å R, Kristinsson SY*, et al.* Risk for Arterial and Venous Thrombosis in Patients With Myeloproliferative Neoplasms: A Population-Based Cohort Study. *Ann Intern Med*. 2018; **168**: 317-25.

25 Bohne LJ, Johnson D, Rose RA, Wilton SB, Gillis AM. The Association Between Diabetes Mellitus and Atrial Fibrillation: Clinical and Mechanistic Insights. *Frontiers in Physiology*. 2019; **10**.

26 Huxley RR, Lopez FL, Folsom AR, Agarwal SK, Loehr LR, Soliman EZ*, et al.* Absolute and Attributable Risks of Atrial Fibrillation in Relation to Optimal and Borderline Risk Factors. *Circulation*. 2011; **123**: 1501-08.

27 Gecht J, Tsoukakis I, Kricheldorf K, Stegelmann F, Klausmann M, Griesshammer M*, et al.* Kidney Dysfunction Is Associated with Thrombosis and Disease Severity in Myeloproliferative Neoplasms: Implications from the German Study Group for MPN Bioregistry. *Cancers (Basel)*. 2021; **13**.

28 Poulin EC, Thibault C. Laparoscopic splenectomy for massive splenomegaly: operative technique and case report. *Can J Surg*. 1995; **38**: 69-72.

29 Wagstaff AJ, Overvad TF, Lip GY, Lane DA. Is female sex a risk factor for stroke and thromboembolism in patients with atrial fibrillation? A systematic review and meta-analysis. *QJM*. 2014; **107**: 955-67.

30 Carobbio A, Thiele J, Passamonti F, Rumi E, Ruggeri M, Rodeghiero F*, et al.* Risk factors for arterial and venous thrombosis in WHO-defined essential thrombocythemia: an international study of 891 patients. *Blood*. 2011; **117**: 5857-59.

31 Senzolo M, Sartori MT, Lisman T. Should we give thromboprophylaxis to patients with liver cirrhosis and coagulopathy? *HPB*. 2009; **11**: 459-64.

32 Folsom AR, Lutsey PL, Roetker NS, Rosamond WD, Lazo M, Heckbert SR*, et al.* Elevated hepatic enzymes and incidence of venous thromboembolism: a prospective study. *Annals of Epidemiology*. 2014; **24**: 817-21.e2.

33 Verbeek TA, Stine JG, Saner FH, Bezinover D. Hypercoagulability in End-stage Liver Disease: Review of Epidemiology, Etiology, and Management. *Transplantation Direct*. 2018; **4**: e403.

34 Lisman T, Leebeek FWG, De Groot PG. Haemostatic abnormalities in patients with liver disease. *Journal of Hepatology*. 2002; **37**: 280-87.

35 Kreher S, Ochsenreither S, Trappe RU, Pabinger I, Bergmann F, Petrides PE*, et al.* Prophylaxis and management of venous thromboembolism in patients with myeloproliferative neoplasms: consensus statement of the Haemostasis Working Party of the German Society of Hematology and Oncology (DGHO), the Austrian Society of Hematology and Oncology (ÖGHO) and Society of Thrombosis and Haemostasis Research (GTH e.V.). *Ann Hematol*. 2014; **93**: 1953-63.

36 Carobbio A, Finazzi G, Guerini V, Spinelli O, Delaini F, Marchioli R*, et al.* Leukocytosis is a risk factor for thrombosis in essential thrombocythemia: interaction with treatment, standard risk factors, and Jak2 mutation status. *Blood*. 2007; **109**: 2310-13.

37 Landolfi R, Di Gennaro L, Barbui T, De Stefano V, Finazzi G, Marfisi R*, et al.* Leukocytosis as a major thrombotic risk factor in patients with polycythemia vera. *Blood*. 2007; **109**: 2446-52.

38 Falanga A, Marchetti M, Vignoli A, Balducci D, Barbui T. Leukocyte-platelet interaction in patients with essential thrombocythemia and polycythemia vera. *Exp Hematol*. 2005; **33**: 523-30.

39 Casini A, Fontana P, Lecompte TP. Thrombotic complications of myeloproliferative neoplasms: risk assessment and risk-guided management. *Journal of Thrombosis and Haemostasis*. 2013; **11**: 1215-27.

40 Buxhofer-Ausch V, Gisslinger H, Thiele J, Gisslinger B, Kvasnicka H-M, Müllauer L*, et al.* Leukocytosis as an important risk factor for arterial thrombosis in WHO-defined early/prefibrotic myelofibrosis: An international study of 264 patients. *American Journal of Hematology*. 2012; **87**: 669-72.

41 Finazzi G, Carobbio A, Thiele J, Passamonti F, Rumi E, Ruggeri M*, et al.* Incidence and risk factors for bleeding in 1104 patients with essential thrombocythemia or prefibrotic myelofibrosis diagnosed according to the 2008 WHO criteria. *Leukemia*. 2012; **26**: 716-19.

42 Koschmieder S. How I Manage Thrombotic/Thromboembolic Complications in Myeloproliferative Neoplasms. *Hämostaseologie*. 2020; **40**: 047-53.
